# VEGF and VEGFR2 bind to similar pH-sensitive sites on fibronectin, exposed by heparin-mediated conformational changes

**DOI:** 10.1016/j.jbc.2021.100584

**Published:** 2021-03-24

**Authors:** Mattia Usuelli, Timmy Meyer, Raffaele Mezzenga, Maria Mitsi

**Affiliations:** Laboratory of Food and Soft Materials, Department of Health Sciences and Technology, ETH Zurich, Zurich, Switzerland

**Keywords:** vascular endothelial growth factor (VEGF), VEGFR2, fibronectin, extracellular matrix, angiogenesis, heparin, hypoxia, acidic, surface plasmon resonance (SPR), AIC, Akaike’s information criterion, BSA, bovine serum albumin, CD, circular dichroism, ECD, extracellular domain, NHS, N-hydroxysuccinimide, RTK, receptor tyrosine kinase, RU, resonance units, SPR, surface plasmon resonance, VEGF, vascular endothelial growth factor, VEGFR2, VEGF receptor 2

## Abstract

Physical interactions between vascular endothelial growth factor (VEGF), a central player in blood endothelial cell biology, and fibronectin, a major fibrillar protein of the extracellular matrix, are important determinants of angiogenic activity in health and disease. Conditions signaling the need for new blood vessel growth, such as hypoxia and low extracellular pH, increase VEGF–fibronectin interactions. These interactions can be further fine-tuned through changes in the availability of the VEGF-binding sites on fibronectin, regulated by conformational changes induced by heparin and heparan sulfate chains within the extracellular matrix. These interactions may alter VEGF bioavailability, generate gradients, or alter the way VEGF is recognized by and activates its cell-surface receptors. Here, using equilibrium and kinetic studies, we discovered that fibronectin can also interact with the extracellular domain of the VEGF receptor 2 (VEGFR2). The VEGFR2-binding sites on fibronectin show great similarity to the VEGF-binding sites, as they were also exposed upon heparin-induced conformational changes in fibronectin, and the interaction was enhanced at acidic pH. Kinetic parameters and affinities for VEGF and VEGFR2 binding to fibronectin were determined by surface plasmon resonance measurements, revealing two populations of fibronectin-binding sites for each molecule. Our data also suggest that a VEGF/VEGFR2/fibronectin triple complex may be formed by VEGF or VEGFR2 first binding to fibronectin and subsequently recruiting the third binding partner. The formation of such a complex may lead to the activation of distinct angiogenic signaling pathways, offering new possibilities for clinical applications that target angiogenesis.

Fibronectin is a major protein of the extracellular matrix, with the ability to interact with a multitude of cell surface and matrix components such as integrins, collagen, and heparan sulfate proteoglycans, as well as many growth factors (1, 2). It plays a crucial role in angiogenesis, the formation of new blood vessels from the preexisting vasculature. Fibronectin null mice die early during embryonic development because of severe vascular defects (3). Furthermore, in many *in vivo* and *in vitro* systems of sprouting angiogenesis, fibronectin is essential for tip cell migration (4, 5, 6), suggesting that it acts as substrate for the growth of the newly formed vessels. Additionally, fibronectin can bind vascular endothelial growth factor (VEGF), a major regulator of angiogenesis, and directly influence its signaling output (7, 8, 9).

Alternative splicing generates several VEGF isoforms, containing a basic domain of increasing length, which interacts with heparan sulfate proteoglycans (10). Traditionally, long VEGF isoforms (*e.g.*, VEGF_206_) with high heparin affinity have been considered matrix-bound, whereas the shorter isoforms (*e.g.*, VEGF_121_), lacking the heparin-binding domain, have been considered completely soluble. Early studies showed that each VEGF isoform has the ability to stimulate angiogenesis *in vivo*, but the morphology and density of the resulting vascular network were aberrant unless the correct balance between all isoforms was maintained (11). However, heparan sulfate proteoglycans are not the only extracellular matrix components interacting with VEGF. Fibronectin is a major binding partner for VEGF isoforms that are considered soluble (VEGF_121_) or exhibiting medium affinity for heparan sulfate proteoglycans (VEGF_165_) (7, 12, 13). Thus, when considering VEGF function, our definition of matrix-bound VEGF should be expanded to include interactions with fibronectin as well as heparan sulfate proteoglycans.

Earlier studies showed that the VEGF-binding sites on fibronectin are cryptic and become exposed upon conformational changes induced by heparin (7). The mechanism of heparin action in this case is fundamentally different from the known allosteric effects of heparin on other proteins, such as antithrombin and FGF, as it does not involve the formation of a ternary complex between VEGF, fibronectin, and heparin (14). Instead, the fast kinetics of the heparin–fibronectin interaction allow for a structural catalysis to take place, whereby substoichiometric amounts of heparin, given enough time, can change the conformation of fibronectin within the entire matrix, by sequential binding to and releasing from neighboring fibronectin molecules. In a physiologic setting, this could be observed locally in the extracellular matrix through the action of heparan sulfate proteoglycans or alternatively, through heparin secreted by mast cells upon inflammation or any other appropriate stimulus. Consequently, such heparin/heparan sulfate-mediated changes in fibronectin conformation within the extracellular matrix will increase VEGF binding and alter its angiogenic potential.

The fact that VEGF binding to the extracellular matrix is an important determinant of its biological output (15, 16, 17) can be partly explained by indirect effects, such as VEGF sequestration in the matrix that alters its bioavailability and generates gradients that influence cellular response (18, 19). However, it has been shown that matrix-bound VEGF, at the same effective amounts as soluble VEGF, in the absence of gradients activates distinct downstream signaling pathways (20, 21). VEGF signals upon binding to cell-surface cognate receptors (VEGFRs), which belong to the family of Receptor Tyrosine Kinases (RTKs) and they regulate a large number of physiological processes in vascular biology (22). There are three known isoforms of VEGFR encoded by different genes: VEGFR1, VEGFR2, and VEGFR3. They are mainly expressed in endothelial cells and, although they share a similar overall architecture, they have significant differences in cellular localization, ligand specificity, structure, and function. VEGFR1 seems to act mostly as a decoy receptor, regulating ligand availability and the signaling strength of VEGFR2, while VEGFR3 functions are limited almost exclusively to the lymphatic system (23). VEGFR2, on the other hand, is the major player in blood endothelial cell biology; it is considered one of the central players in regulating angiogenesis in a number of physiological and pathological conditions, such as embryonic development, wound healing, and cancer growth and metastasis (24).

Canonical VEGF signal transduction proceeds *via* dimerization of VEGFR2 and propagation of conformational changes, which are not yet fully understood, but eventually lead to a precise juxtaposition and activation of the intracellular kinase domain of the receptors (22). Subsequently, a series of tyrosine residues in the intracellular part of VEGFR2 become phosphorylated, initiating various signaling cascades. There is evidence that VEGFR2 signaling is modulated by various coreceptors, including integrins that interact with extracellular matrix components. Thus, matrix-bound VEGF could recruit different coreceptors to the VEGF/VEGFR2 complex, leading to distinct VEGFR2 activation patterns (25, 26, 27, 28). Indeed, the distinct phosphorylation profile of VEGFR2 activated by immobilized VEGF involves β_1_ integrin-induced VEGFR2 clustering (20) and numerous studies suggest extensive cross talk between α_v_β_3_ and α_5_β_1_ integrins—the major fibronectin receptors—and VEGFR2 (29). Thus, complex formation between VEGF and fibronectin could bring together VEGFR and integrins into a large signaling complex leading to distinct pathway activation.

The early recognition of the importance of angiogenesis for cancer growth and metastasis (30), led to the development of several cancer therapeutic approaches that target the VEGF/VEGFR2 signaling axis (31). However, despite great promise from initial studies, their clinical outcome has been at best meagre, accompanied by severe side effects (32). Although the ineffectiveness of antiangiogenic therapies cannot be ascribed to a single factor, considering the complexity of the biology and clinical manifestation of cancer, it certainly highlights the need to draw a more complete picture of the mechanisms underlying VEGFR2 activation and signaling. In particular, targeting specifically matrix-bound VEGF and the interactions that hold the VEGF/VEGFR2 complex together may offer exciting new opportunities for the design of more specific and effective drugs (33). Therefore, it is important for both basic research and clinical applications to understand the dynamic interactions between VEGF, VEGFR2, and fibronectin.

In this study, we discovered that the catalytic effect of heparin on fibronectin structure exposes also binding sites for the extracellular domain (ECD) of VEGFR2. Equilibrium and kinetic studies revealed that ECD binding and VEGF binding to fibronectin followed a very similar pattern and were enhanced at acidic pH. We found that the affinity of ECD for fibronectin was higher than that of VEGF. Furthermore, VEGF and ECD could bind simultaneously to fibronectin. Such binding events could influence the activation of VEGF/VEGFR2 complexes, as well as the formation of higher-order complexes with other cell-surface receptors and extracellular matrix proteins, regulating angiogenesis in health and disease.

## Results

### Equilibrium binding by enzyme-linked immunoassay (ELISA)-based experiments

To study the interactions between fibronectin and the angiogenic factors VEGF_165_ (termed VEGF for simplicity) and ECD in a simple and fast way that would not require protein labeling, we developed an ELISA-based solid-phase binding assay. Fibronectin was adsorbed on 96-well plates and allowed to interact with the ligand (VEGF or ECD) until equilibrium was reached. The fraction of the ligand that was specifically bound to fibronectin was then extracted with a solution of high ionic strength (5 M NaCl), adsorbed onto a second 96-well plate, and detected using a primary antibody against the ligand and a corresponding HRP-labeled secondary antibody. The signal was detected by luminescence. The ligand extraction step was necessary because of the high levels of VEGF nonspecific binding, which could not be reduced by blocking agents either added in the binding buffer or adsorbed on the plate. The steps for the assay optimization are described in Figs. S1–S6.

**Figure 1:**
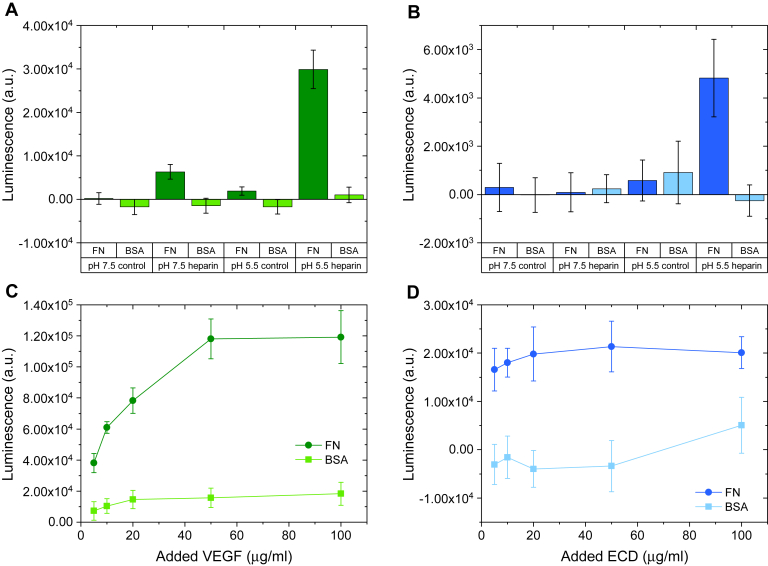
**Acidic pH and pretreatment of fibronectin with heparin increase VEGF and ECD binding.***A* and *B*, Fibronectin (*dark green/blue bars*) or bovine serum albumin (BSA) (*light green/blue bars*) was adsorbed on 96-well polystyrene plates (20 μg/ml; 50 μl/well) overnight at 4 °C. The plate was incubated for 1 h on ice with 5 μg/ml VEGF (*A*) or ECD (*B*) in binding buffer (150 mM NaCl, 25 mM Hepes) at pH 7.5 or 5.5, in the absence (control) or presence (heparin) of pretreatment of the adsorbed proteins with 100 μg/ml heparin in PBS (1 h on ice). Bound VEGF or ECD was extracted with 5 M NaCl, 25 mM Hepes, pH 7.5 for 1 h on ice, readsorbed on a second plate, and detected by ELISA with an anti-His primary antibody (1:1000) and an HRP-labeled secondary antibody (1:2000). Both antibody incubations were performed in ELISA blocking buffer (1 mg/ml BSA + 0.05% Tween20 in PBS). Samples were measured in quadruplicate, and the data are presented as mean values ± standard deviation. *C* and *D*, Fibronectin (*dark green/blue lines*) or BSA (*light green/blue lines*) was adsorbed on 96-well polystyrene plates (20 μg/ml; 50 μl/well) overnight at 4 °C. The plate was incubated for 1 h on ice with increasing concentrations of VEGF (*C*) or ECD (*D*) in binding buffer (150 mM NaCl, 25 mM Hepes) at pH 5.5 in the presence of pretreatment of the adsorbed proteins with 100 μg/ml heparin in PBS (1 h on ice). VEGF and ECD extraction and detection were performed as in (*A*) and (*B*). Samples were measured in quadruplicate, and the data are presented as mean values ± standard deviation.

Using this assay, we evaluated the binding of VEGF and ECD to surface-immobilized fibronectin. VEGF binding followed the same pattern as previously reported, with higher binding occurring at acidic pH only after fibronectin was treated with heparin (Fig. 1*A*). The measured values reflect the VEGF fraction specifically bound to fibronectin, since no VEGF was extracted from the naked substrate or from adsorbed bovine serum albumin (BSA). Interestingly, a very similar binding pattern was observed with ECD (Fig. 1*B*). For both ligands, significant binding was observed at pH 5.5 after incubating the surface-immobilized fibronectin with heparin. Thus, we chose this condition to further analyze the protein interactions.

**Figure 2:**
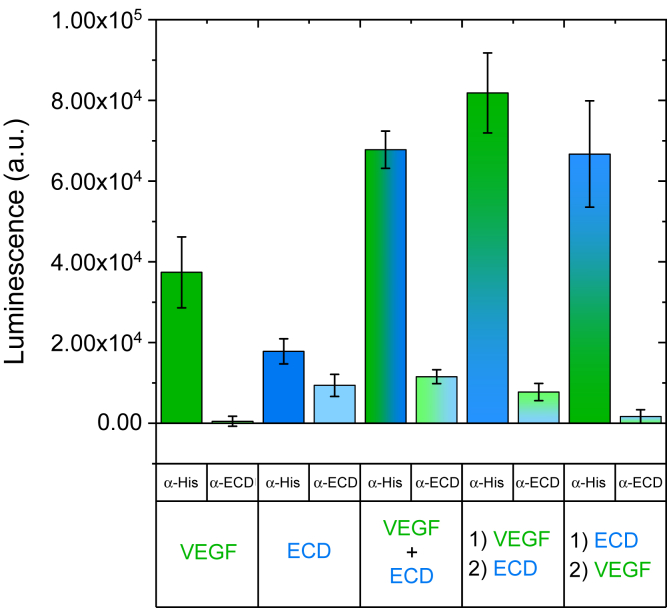
**VEGF and ECD can bind simultaneously to fibronectin.** Fibronectin was adsorbed on 96-well polystyrene plates (20 μg/ml; 50 μl/well) overnight at 4 °C. The plate was incubated for 1 h on ice with 50 μg/ml of VEGF or ECD alone or in combination (VEGF + ECD). Additionally, some wells were treated first with 50 μg/ml of VEGF or ECD for 1 h, followed by a second 1 h incubation with 50 μg/ml ECD or VEGF, respectively (conditions labeled as ‘1) VEGF 2) ECD’ and ‘1) ECD 2) VEGF’, respectively). Ligand binding was studied in binding buffer (150 mM NaCl, 25 mM Hepes) at pH 5.5 after pretreatment of the adsorbed fibronectin with 100 μg/ml heparin in PBS (1 h on ice). Bound VEGF and/or ECD were extracted with 5 M NaCl, 25 mM Hepes, pH 7.5 for 1 h on ice, readsorbed on a second plate, and detected by ELISA with an anti-His or an anti-VEGFR2 primary antibody (1:1000) and an HRP-labeled secondary antibody (1:2000). Both antibody incubations were performed in ELISA blocking buffer (1 mg/ml bovine serum albumin + 0.05% Tween20 in PBS). Samples were measured in quadruplicate, and the data are presented as mean values ± standard deviation.

Dose–response experiments showed that ECD binding to surface-immobilized fibronectin reached saturation at significantly lower concentrations than those for VEGF, suggesting that ECD binds to fibronectin with higher affinity than VEGF (Fig. 1, *C* and *D*). This conclusion is further reinforced if we consider that the binding was performed with equal masses for the two ligands, which, given the difference in molecular weight between VEGF (45 kDa) and ECD (83 kDa), means lower molarity for ECD. We did not attempt to calculate affinities at this stage, because of the many intermediate assay steps between the binding event and the final measurement. However, we observed significantly lower luminescence values for ECD than for VEGF (Fig. 2). These differences could originate from different recognition of VEGF and ECD by the primary antibody and/or different degrees of adsorption of the two proteins. Indeed, calibration curves revealed different signals for the same amount of VEGF and ECD, which depended on both the ionic strength of the buffer and the protein concentration (Fig. S6). Taken all this into account, it appears that ECD possesses higher affinity for fibronectin than VEGF, but the absolute amount of VEGF and ECD bound to fibronectin cannot be determined by this experimental setup.

Since VEGF and ECD bind to fibronectin following a very similar pattern, we wanted to elucidate whether they share the same binding sites and if their binding is mutually exclusive. To do so, we compared the combined binding of VEGF and ECD to fibronectin when they were added simultaneously or sequentially, using an anti-His antibody that can recognize both ligands (as they both contain a His-tag; see Experimental Procedures) or an anti-VEGFR2 antibody that recognizes specifically ECD (Fig. 2). We confirmed that both the anti-His and anti-VEGFR2 antibodies could detect their respective ligands when bound individually to fibronectin. When both VEGF and ECD were added to fibronectin, the signal detected by the anti-His antibody was always higher than if VEGF or ECD was added alone, irrespectively of the order of ligand addition, suggesting that their binding to fibronectin is not mutually exclusive. The anti-VEGFR2 antibody detected the presence of ECD under all conditions, except when ECD was added before VEGF. Since no binding scenario predicts the complete absence of ECD under this condition (Fig. S7), this result suggests that when fibronectin-bound ECD is allowed to interact with VEGF as well, the epitope for this particular antibody is masked. The only possibility consistent with all these observations is that VEGF and ECD are likely to share binding sites on fibronectin, and when bound to fibronectin, they retain their ability to recognize each other and lead to the formation of a VEGF/ECD/fibronectin triple complex (Fig. S7).

**Figure 3:**
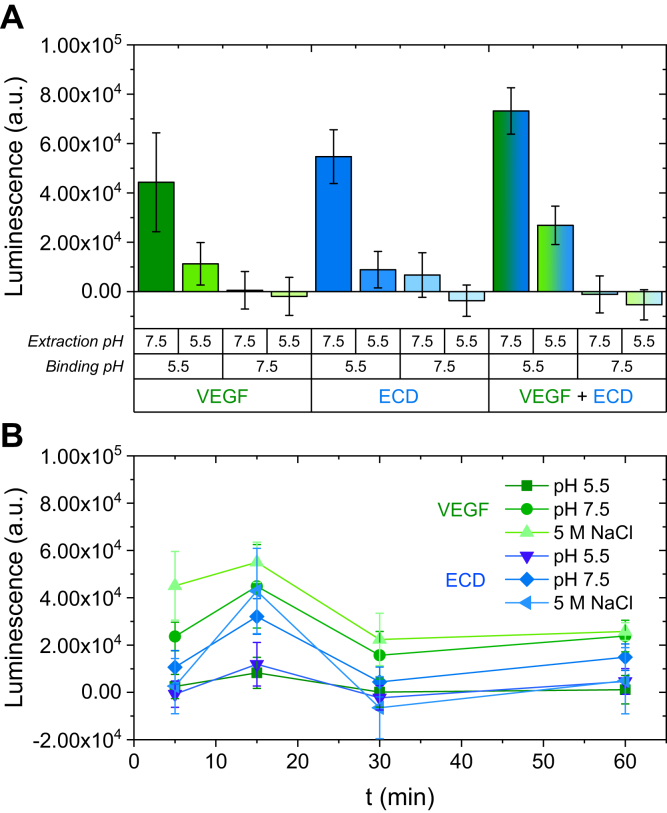
**Switching the pH from acidic (5.5) to neutral (7.5) is sufficient to release fibronectin bound VEGF and ECD.***A*, Fibronectin was adsorbed on 96-well polystyrene plates (10 μg/ml; 50 μl/well) overnight at 4 °C. The plate was incubated for 1 h on ice with 50 μg/ml of VEGF (*green*) or ECD (*blue*) alone or in combination (VEGF + ECD) in binding buffer (150 mM NaCl, 25 mM Hepes) at pH 7.5 or 5.5 after pretreatment of the adsorbed fibronectin with 100 μg/ml heparin in PBS (1 h on ice). Bound VEGF and ECD were extracted with binding buffer (150 mM NaCl, 25 mM Hepes) at pH 7.5 or 5.5, for 1 h on ice, readsorbed on a second plate, and detected by ELISA with an anti-His primary antibody (1:1000) and an HRP-labeled secondary antibody (1:2000). Both antibody incubations were performed in ELISA blocking buffer (1 mg/ml bovine serum albumin + 0.05% Tween20 in PBS). Samples were measured in quadruplicate, and the data are presented as mean values ± standard deviation. *B*, Fibronectin was adsorbed on 96-well polystyrene plates (10 μg/ml; 50 μl/well) overnight at 4 °C. The plate was incubated for 1 h on ice with 50 μg/ml of VEGF (*green*) or ECD (*blue*) in binding buffer (150 mM NaCl, 25 mM Hepes) at pH 5.5 after pretreatment of the adsorbed fibronectin with 100 μg/ml heparin in PBS (1 h on ice). Bound VEGF and ECD were extracted with binding buffer (150 mM NaCl, 25 mM Hepes) at pH 7.5 or 5.5, or with 5 M NaCl, 25 mM Hepes, pH 7.5 for 5, 15, 30 or 60 min (each time interval in a different set of wells) on ice, readsorbed on a second plate, and detected by ELISA with an anti-His primary antibody (1:1000) and an HRP-labeled secondary antibody (1:2000). Both antibody incubations were performed in ELISA blocking buffer (1 mg/ml bovine serum albumin + 0.05% Tween20 in PBS). Samples were measured in quadruplicate, and the data are presented as mean values ± standard deviation.

Both VEGF and ECD could be released from fibronectin by changing the pH from 5.5 to 7.5, even if the ionic strength of the extraction solution was not increased (Fig. 3*A*). Indeed, gradually increasing the ionic strength of the extraction buffer did not result in higher release (Fig. S8). This suggests that the interaction depends on pH-sensitive amino acids. The release was very fast, occurring within a few minutes after buffer change. Interestingly, longer extraction times led to a decrease in the amount of released VEGF or ECD (Fig. 3*B*), which may be the result of ligand readsorption (Fig. S8).

### Structural determinants of the interactions

**Figure 4:**
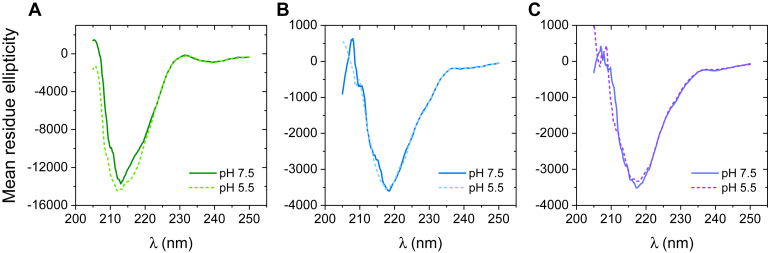
**The secondary structure of VEGF and ECD does not undergo significant changes between neutral and acidic pH.** Circular dichroism spectra were acquired for VEGF (*A*; 0.3 mg/ml), ECD (*B*; 0.3 mg/ml), and VEGF/ECD complex (*C*; 0.5 mg/ml) in binding buffer (150 mM NaCl, 25 mM Hepes) at pH 7.5 (*solid lines*) and pH 5.5 (*dashed lines*) at 4 °C. The data are presented in mean residue ellipticity units (deg∗cm^2^/dmol).

Circular dichroism (CD) spectra of VEGF and ECD were acquired at pH 7.5 and pH 5.5 (Fig. 4). The ionic strength of the buffer prevented data collection below 205 nm, and at low ionic strength buffers, the structure of VEGF was altered, suggesting significant unfolding (Fig. S9). Therefore, the spectra could not be subjected to structure deconvolution with certainty, especially, since both VEGF and ECD are beta-sheet dominated and the CD spectra of beta-sheet proteins show significant variability, rendering their deconvolution problematic (34). However, the spectra at pH 7.5 and 5.5 were very similar, suggesting that the pH does not cause a major change in the protein secondary structure. If there is a pH-sensitive conformational change of VEGF and ECD, it should be slight or local. Therefore, it is more likely that the increased binding observed at pH 5.5 would depend on the protonation state of critical amino acids in the binding site rather than large conformational changes. We decided to further study this point, by computing the pH dependance of the protonation state of surface amino acids for both the ligands (VEGF and ECD) and the domain of fibronectin where the binding is likely to happen (FNIII 12–14) (7).

Based on the known structures of VEGF and ECD, we calculated the pKa values of surface-exposed amino acids (35), and we identified several residues with a pKa value between 5.5 and 7.5, whose protonation state would change between these two pH values (Table 1) and thus, could belong to the fibronectin-binding sites on each molecule. In the case of ECD, most of the candidate residues were His residues, which often function as pH sensors (36, 37, 38). Whereas for ECD almost all candidate residues seem to be free of interactions with the ligand, the majority of the pH-sensitive residues on VEGF were on the receptor-binding domain and were involved in interactions in the VEGF dimer or between VEGF and the receptor, leaving free of interactions only a His residue at the C-terminal domain of VEGF. However, since full-length structures are lacking for both VEGF and ECD, these results should be interpreted with caution since prediction of pKa values is very sensitive to the local environment of the amino acid (39, 40). Because of the pH sensitivity of His residues, we would like to mention that experiments with nontagged VEGF conducted in an earlier study showed the same binding profile (7). Moreover, His-tagged VEGF-E showed no binding to heparin-treated fibronectin at acidic pH (data not shown). These observations support the conclusion that the His tag in the recombinant proteins used in this study is unlikely to affect the enhanced binding observed at acidic pH.

**Table 1:** Surface-exposed amino acid residues on VEGF and ECD predicted to change protonation state between pH 7.5 and 5.5

	Protein domain	Residue	pKa	Protonation probability		Free of interactions
				pH 5.5	pH 7.5	
VEGF	Receptor-binding domain	Arg23	6.82	0.994	0.074	No
		Lys84	6.46	0.973	0.017	No
		His86	6.33	0.963	0.012	No
		Glu93	6.37	0.967	0.015	No
	Heparin-binding domain	His125	5.96	0.847	0.002	Yes
ECD	Domain1–domain2 linker	Arg122	6.46	0.980	0.020	Yes
		His133	6.52	0.980	0.020	Yes
	Domain2	Arg176	6.35	0.970	0.010	Yes
		Glu201	6.37	0.970	0.010	Yes
	Domain3	His232	6.33	0.960	0.010	Yes
		Glu235	6.89	1.000	0.100	Yes
		Arg249	6.20	0.930	0.010	Yes
		His267	6.21	0.930	0.010	Yes
		His269	6.44	0.970	0.020	Yes
		Asp295	6.80	0.990	0.070	Yes
	Domain3–domain4 linker	Arg323	6.31	0.960	0.010	No
		His325	6.69	0.990	0.040	Yes
	Domain4	His375	6.25	0.950	0.010	Yes
		His381	6.25	0.950	0.010	Yes
		His411	6.72	0.990	0.040	Yes
	Domain5	His454	6.13	0.870	0.010	Yes
		His455	6.00	0.770	0.010	Yes
		His457	6.46	0.940	0.030	Yes
		His546	7.02	1.000	0.140	Yes

Structure-based predictions of pKa values of individual amino acid residues at the surface of VEGF and ECD were performed with the DelPhiPKa web server, using the known high-resolution structures of VEGF and ECD fragments (pdb files used: 2VPF and 2VGH for VEGF and 3V2A, 2X1W, 2X1X, 3S35, 3S36, 3S37, 5OYJ, and 3KVQ for ECD). It is also indicated whether the side chains of the candidate amino acids are free from known interactions (hydrogen bonds and salt bridges) with other parts of the protein or holding together the VEGF/ECD complex.

As mentioned above, we performed a similar analysis on the C-terminal 40 kDa fragment of fibronectin encompassing domains FNIII 12–14, which was identified earlier as the VEGF-binding site on fibronectin (7, 8). Interestingly, no pH-sensitive residues with a pKa value between 5.5 and 7.5 were identified in this region. We run the analysis using two different conformations reported for this fibronectin domain (pdb codes: 1FNH and 3R8Q), obtaining almost identical results: 22 acidic residues (pKa < 4), 32 basic residues (pKa > 10), and no exposed pH-sensitive residues. This suggests that, after being exposed by the heparin action, this binding site on fibronectin is not regulated further by changing the local pH. Instead, it remains always available for binding, which occurs only when key amino acids in the ligand become protonated at acidic pH.

### Mechanistic insights on the interactions by SPR kinetic experiments

To further understand mechanistically the interactions between fibronectin and VEGF/ECD, we followed the kinetics of the interactions by surface plasmon resonance (SPR). Fibronectin was immobilized *via* N-hydroxysuccinimide (NHS) chemistry on the surface of the carboxymethyldextran hydrogel of the biosensor chip (Fig. S10). VEGF or ECD was added in the mobile phase under flow. Based on the results of the equilibrium studies, all experiments were performed at pH 5.5. Binding in the absence of any heparin treatment of the surface-immobilized fibronectin was minimal, even at a high fibronectin density (Fig. S11). We tested the effect of injecting 1 mg/ml BSA into both flow cells, which could act as a blocking protein, reduce nonspecific binding of VEGF to both flow cells, and unmask specific associations between VEGF and the immobilized fibronectin. However, the low levels of VEGF binding persisted. Interestingly, BSA injection stabilized the sensogram values for background VEGF binding after repeated injection and regeneration cycles (Fig. S12) and was, therefore, employed in all subsequent experiments. Heparin treatment of the immobilized fibronectin was thus necessary in order to observe any appreciable binding. Based on the resulting resonance units (RU) values following heparin treatment, we chose an intermediate fibronectin density (2 μg/ml) to get values within the recommended range for SPR experiments (Fig. S11).

**Figure 5:**
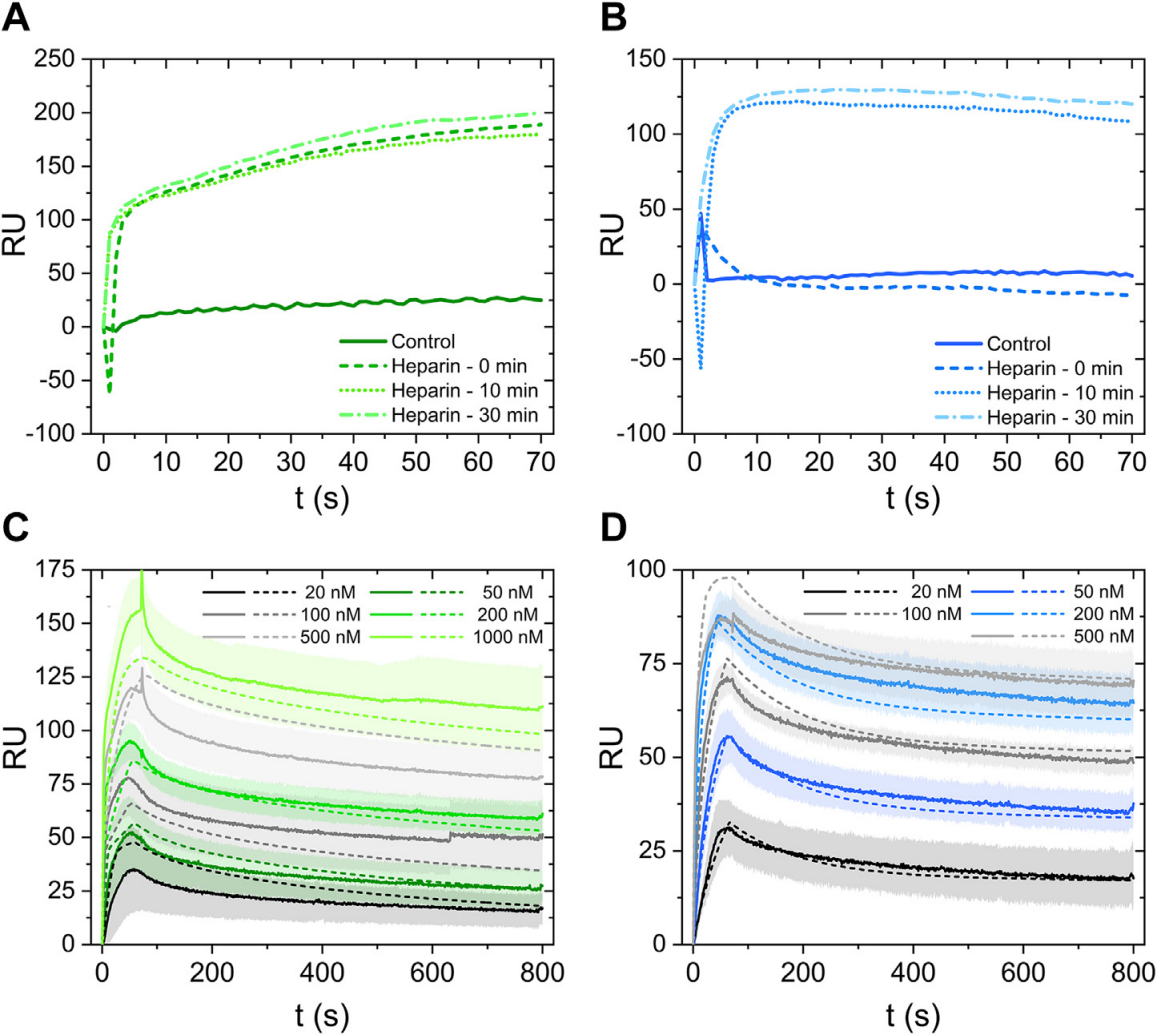
**Direct monitoring of interactions between fibronectin and VEGF or ECD by SPR.***A* and *B*, VEGF (*A*) or ECD (*B*) (60 μl of 1000 nM) was injected at a flow rate of 50 μl/min on a chip with 2 μg/ml immobilized fibronectin (140 ng) in the absence (control) or presence of a heparin pretreatment of the immobilized fibronectin, followed by heparin dissociation for 0 min, 10 min, or 30 min. *C* and *D*, Heparin (50 μl of 1 μg/ml) was injected at a flow rate of 50 μl/min on a chip with 2 μg/ml immobilized fibronectin (140 ng). Following association, heparin was allowed to dissociate for 10 min, and immediately afterward, VEGF (*C*) or ECD (*D*) was injected (60 μl at 50 μl/min) at different concentrations: 20 nM, 50 nM, 100 nM, 200 nM, 500 nM, and 1000 nM for VEGF and 20 nM, 50 nM, 100 nM, 200 nM, and 500 nM for ECD (the color code is explained in the figure). Mean values from triplicate measurements are shown, with the shaded area representing the standard deviation. Following association, VEGF and ECD were allowed to dissociate for 720 s. The different VEGF/ECD concentrations were injected following a random order in order to ensure that the measurements were free of systematic errors, and the progressive increase in VEGF/ECD binding with increasing concentration reflected the true binding. The surface was regenerated with 2 M NaCl and 0.05 N NaOH after every VEGF/ECD binding cycle. The experimental data (*solid lines*) were fitted to a two-sites model (*dashed lines*) considering mass transfer for VEGF (*C*) and rapid mixing for ECD (*D*).

To test the action of heparin in the SPR setup, ∼10x molar excess of heparin was injected prior to the binding assay, was allowed to associate with the surface-immobilized fibronectin for 1 min and then dissociate for increasing periods of time (0, 10, or 30 min) by changing accordingly the flow rate (Fig. 5, *A* and *B*). This experimental design was based on a previous study, describing the catalytic mechanism by which heparin mediates the conformational change on fibronectin that exposes the VEGF-binding sites (14). According to this model, fibronectin–heparin interactions are transient and characterized by multiple rebinding and release events, resulting in a low amount of bound heparin at equilibrium. In the SPR setup, heparin rebinding could occur during the long dissociation phase, increasing the contact time between heparin and fibronectin and resulting in maximal conversion of fibronectin to the open form, exposing the binding sites. At the same time, the longer dissociation times would allow for a more complete heparin release, thus, minimizing the amount of heparin present on the surface-immobilized fibronectin during the binding assay with VEGF/ECD. Unfortunately, an estimation of the amount of heparin bound to fibronectin directly from the SPR sensograms was not possible, because of the low signal generated by this amount of heparin (Figs. S13 and S14), further demonstrating the low levels of heparin binding to fibronectin. However, the VEGF sensograms show that the duration of the dissociation step following heparin treatment did not affect VEGF binding (Fig. 5*A*). This supports the idea that a 1-min association time was sufficient for this amount of heparin (1 μg/ml) to convert the immobilized fibronectin to the open conformation (this structural rearrangement has been reported earlier to be fast (14)) and that the subsequent VEGF binding occurs on fibronectin and not on any residual heparin remaining bound to fibronectin. Interestingly, ECD binding stabilized only after at least 10 min of heparin dissociation (Fig. 5*B*). If no dissociation was performed following heparin treatment, there was no ECD binding. This would be explained if ECD shared binding sites with heparin, and in order to observe appreciable binding, heparin must be completely released. Based on these results, we performed all further experiments after heparin treatment of the surface-immobilized fibronectin by 1 min association, followed by 10 min dissociation.

To extract kinetic rates, we collected association and dissociation data with a concentration series of VEGF or ECD (Fig. 5, *C* and *D*). Already a visual inspection of the binding curves, prior to any attempt to fit a model for the macromolecular interaction, reveals certain characteristics. First, there was a very fast initial burst during the association phase, especially evident at higher VEGF/ECD concentrations, followed by a slower increase. Second, dissociation was slow, resulting in a significant amount of ligand remaining bound at the end of the experiment, which was increased with VEGF/ECD concentration.

**Figure 6:**
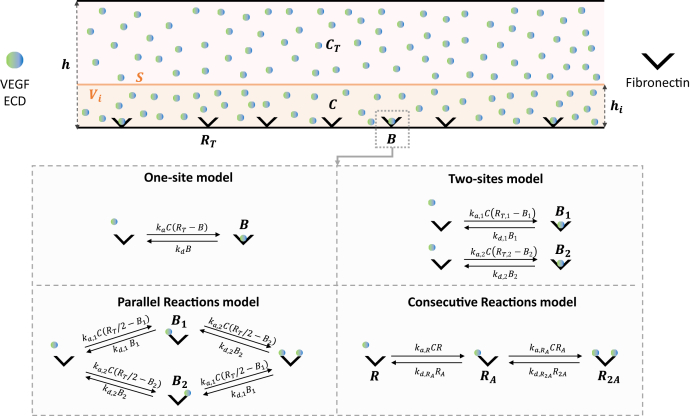
**Modeling of interactions between fibronectin and VEGF or ECD during an SPR experiment.** The SPR experimental setup was modeled using the two-compartment model considering (1) a flowing bulk phase with a time-invariant concentration (*C*_*T*_) of ligands (VEGF or ECD) and (2) a narrow volume slit (*V*_*i*_) in the proximity of the surface (*S*) where receptors (fibronectin) have been immobilized at an initial concentration *R*_*T*_. Ligand molecule exchange occurs between the flowing bulk phase and *V*_*i*_ (mass transfer), as well as between *V*_*i*_ and *S* (binding/unbinding events). For simplicity, the two monomeric chains of a fibronectin molecule are considered to act independently, and the surface-immobilized receptors depicted correspond to each monomeric chain and not to the complete fibronectin dimer. Four different models were used to describe ligand–receptor interactions, generating ligand-bound (*B*) receptor molecules: the one-site model, assuming one ligand binding site per receptor; the two-sites model, assuming a heterogeneous receptor population composed of two types of molecules, each with a single binding site with distinct association/dissociation kinetics; the parallel reactions model, assuming that each receptor contains two ligand-binding sites that act independently; and the consecutive reactions model, assuming that each receptor contains two allosterically linked ligand binding sites, where a second ligand can bind only after the first site has already been engaged. From a modeling perspective, we consider the concentration of binding sites for the one-site (*B*), two-sites (*B*_*1*_ and *B*_2_), and parallel reactions (*B*_*1*_ and *B*_2_) models, and the concentration of the various receptor molecular species (*R*, *R*_*A*_, and *R*_*2A*_) for the consecutive reactions model.

A conventional one-site model (Fig. 6) failed to fit the data (section 6 in Experimental Procedures; Figs. S15 and S16), even when considering mass transfer effects. Given the observed slow dissociation, we considered a model whereby the ligand upon dissociation from one receptor molecule performs a random walk across the surface and rebinds to neighboring receptor molecules multiple times before diffusing back to the bulk phase (41). However, this model also failed to capture the binding behavior, as it could not account for the fact that the dissociation rate depended on the VEGF/ECD concentration (Fig. S17). We considered also models whereby each monomeric chain of fibronectin possesses two binding sites for VEGF or ECD, which could act independently (parallel reactions model) or affect each other (consecutive reactions model) (Fig. 6). Although the consideration of two binding sites improved somewhat the fitting, there were still significant deviations from the experimental data (Figs. S15 and S16). The model that generated the best fit was the two-sites model, assuming two populations of fibronectin molecules, each possessing a single binding site per monomeric chain that interacts with the ligand (VEGF or ECD) with distinct association/dissociation rate constants (Fig. 5, *C* and *D*; Table 2). Interestingly, only VEGF binding required consideration of mass transfer phenomena for better fitting, which may be related to the faster association rates for VEGF than ECD, rendering the process diffusion limiting. The kinetic parameters governing fibronectin-VEGF/ECD interactions extracted from the two-sites model are summarized in Table 3. Instead of single values, we report intervals, which were either calculated during fitting for the parameters extracted directly from the model or derived from these values for the K_d_ estimates.

**Table 2:** Statistical evaluation of different models describing the interaction between fibronectin and VEGF or ECD

		One-site model		Two-sites model		Parallel reactions model		Consecutive reactions model	
		Rapid mixing	Mass transfer	Rapid mixing	Mass transfer	Rapid mixing	Mass transfer	Rapid mixing	Mass transfer
VEGF	Data points	4800	4800	4800	4800	4800	4800	4800	4800
	Parameters	3	4	6	7	5	6	5	6
	RMS	7.34E+05	8.89E+05	5.17E+05	4.93E+05	7.02E+05	7.71E+05	3.98E+05	3.27E+05
	σ^2^	153	185	108	103	146	161	83	68
	σ	12	14	10	10	12	13	9	8
	AIC	10,493	10,895	9769	9670	10,404	10,602	9221	8814
	Ranking	6	8	4	3	5	7	2	1
ECD	Data points	4000	4000	4000	4000	4000	4000	4000	4000
	Parameters	3	4	6	7	5	6	5	6
	RMS	9.09E+04	4.78E+05	5.02E+04	5.48E+04	7.26E+04	1.27E+05	6.84E+04	5.79E+04
	σ^2^	23	120	13	14	18	32	17	14
	σ	5	11	4	4	4	6	4	4
	AIC	5432	8318	4407	4563	5047	6014	4943	4655
	Ranking	6	8	1	2	5	7	4	3

The experimental data presented on Fig. 5, *C* and *D* for the interaction between fibronectin and VEGF or ECD were fitted with the models shown on Fig. 6, and the fits that generated well-defined solutions (Figs. S15 and S16 and Table S2) were evaluated with measures for absolute fit (RMS and σ^2^) and model parsimony (AIC), as described in the section. Statistical analysis of curve fitting' under Experimental Procedures. The number of data points is lower for ECD than for VEGF, since the curve corresponding to 1000 nM ligand was not considered for the fitting in the case of ECD because the interaction reached saturation already at 500 nM ligand and additionally, the data associated with 1000 nM ECD displayed a large experimental error. The number of parameters for each model is derived from Fig. 6, and the corresponding parameter estimates are shown in Table S2.

**Table 3:** Kinetic parameters describing the interactions between fibronectin and VEGF or ECD

	Site A			Site B			α	R_T_ (mol^1^m^-2^)	k_m_ (m^1^s^-1^)
	k_a_ (M^-1^s^-1^)	k_d_ (s^-1^)	K_d_ (nM)	k_a_ (M^-1^s^-1^)	k_d_ (s^-1^)	K_d_ (nM)			
VEGF	9.0∗10^6^ 3.1∗10^7^	2.9∗10^-3^ 1.0∗10^-4^	0.07 0.6	6.3∗10^4^ 6.7∗10^4^	5.3∗10^-5^ 1.9∗10^-4^	6.5 8.7	0.32 0.34	3.0∗10^-9^ 3.0∗10^-9^	2.2∗10^-6^ 3.7∗10^-6^
ECD	8.8∗10^5^ 1.1∗10^6^	7.0∗10^-3^ 8.2∗10^7^	0.8 2.9	2.1∗10^5^ 2.1∗10^5^	2.8∗10^-5^ 9.2∗10^-5^	0.1 0.4	0.23 0.26	1.2∗10^-9^ 1.2∗10^-9^	

The experimental data presented on Fig. 5, *C* and *D* were fitted with the two-sites model (considering mass transfer for VEGF and rapid mixing for ECD). Association and dissociation rates (k_a_ and k_d_) for each site, the site occupancy α (Site A/Site B), the density of total binding sites (R_T_), and the mass transfer rate (k_m_), when applicable, were extracted from the model. Instead of single values, the lower and upper boundaries of the confidence intervals for each parameter, calculated during fitting, are given. Dissociation affinity constants for each site were calculated by dividing k_d_ by k_a_. The intervals for the K_d_ values were derived from those associated with the k_d_ by k_a_ values according to the formula: for Z = A/B, (ΔZ/Z)^2^ = (ΔA/A)^2^ + (ΔB/B)^2^, where ΔA, ΔB, and ΔZ are the errors associated with quantities A, B, and Z, respectively.

Statistical analysis indicates that the discrepancies observed between experimental and fitted data can be ascribed to experimental error (section Statistical analysis of curve fitting in Experimental Procedures; Figs. S18 and S19; Table S2). Additional uncertainties in parameter estimation could originate from the limited number of experimental points during the initial association burst. However, it is possible that a simple two-sites model fails to describe certain aspects of the macromolecular interactions, such as rebinding events, and it should be considered only as an approximation of the experimental observations.

## Discussion

VEGF binding to the extracellular matrix has been recognized as an important event regulating angiogenesis (2). Previous studies have shown that fibronectin, a major component of the extracellular matrix, possesses cryptic binding sites for VEGF, which become available when fibronectin undergoes a conformational change, catalyzed by heparin and heparan sulfate chains within the extracellular matrix (7, 8, 13). VEGF binding to these sites is enhanced at acidic pH (7, 12). Here, we report for the first time a similar class of heparin-sensitive binding sites on fibronectin that interact with the ECD of VEGFR2, also at acidic pH. VEGFR2 is a major cell-surface receptor for VEGF, and it is possible that interactions between VEGF, VEGFR2, and fibronectin are important for the regulation of VEGF/VEGFR2 signaling, which is critical for angiogenesis (42). In this study, we developed assays in order to characterize these protein–protein interactions by equilibrium and kinetic studies *in vitro*.

For equilibrium studies, we followed an ELISA-based approach, which does not require ligand labeling. This not only increases the versatility and simplicity of the assay, but also circumvents the need for testing the potential effects of labeling on the structure, activity, and stability of the ligand. However, we noticed high levels of nonspecific VEGF binding to the plate surface, which masked the specifically bound VEGF to fibronectin and prevented its direct detection. Several blocking protein-based agents (BSA, egg white albumin, beta-lactoglobulin, gelatin, hemoglobin, and milk) were tested, either by coadsorption on the substrate or by inclusion in the binding buffer, but none could suppress VEGF nonspecific binding consistently. Therefore, we used an indirect approach, whereby we extracted the bound ligand by either increasing the ionic strength of the buffer or changing the pH back to neutral, readsorbed it on another assay plate, and detected it with a typical ELISA. Negative controls with the uncoated substrate or with adsorbed BSA instead of fibronectin confirmed that the extraction step released only the fraction of the ligand that interacted specifically with fibronectin (Fig. 1). In our experiments, we used recombinant VEGF and ECD proteins carrying a His-tag, which allowed the detection of both proteins by the same anti-His primary antibody, rendering the assay uniform. However, the assay worked well also with ligand-specific antibodies (Fig. 2). A drawback of this assay design is the number of intermediate steps before ligand detection (ligand binding, extraction, readsorption, and ELISA), which makes it difficult to extract equilibrium constants for the interactions. Nonetheless, our ELISA-based binding assay can be readily applied to compare the specific binding of different VEGF/VEGFR isoforms, fragments, and mutants, and potentially additional growth factors and cytokines, to fibronectin.

The results of our ELISA-based binding assay show that binding of both VEGF and ECD to surface-adsorbed fibronectin requires opening up of cryptic binding sites on the fibronectin molecules through the catalytic action of heparin and is enhanced at acidic pH. The heparin-exposed VEGF-binding sites have been localized on the 40 kDa C-terminal domain of fibronectin, comprising domains FNIII 12–14 (7, 8, 13). Although it is possible that the heparin-catalyzed structural changes may affect more than one region of fibronectin, our binding experiments with sequential ligand addition suggest that VEGF and ECD may share binding sites on fibronectin, and binding of either one can bring the entire VEGF/VEGFR2 complex in contact with fibronectin (Fig. 2). This is consistent with previous studies showing the ability of fibronectin-bound VEGF to interact with VEGFR2 (21).

Mechanistic insights on the interactions between fibronectin and VEGF/VEGFR2 were gained by SPR kinetic experiments. Similar to the results of the equilibrium studies, binding of either VEGF or ECD to fibronectin at acidic pH was minimal in the absence of any heparin treatment. To achieve the maximum effect of heparin on the conformation of the immobilized fibronectin, while minimizing VEGF or ECD binding to any residual heparin remaining bound to fibronectin, we allowed fibronectin to interact with ∼10x molar excess heparin during a 1-min association phase, followed by a 10-min dissociation phase (Fig. 5). Previous research has shown that heparin–fibronectin interactions are transient and governed by repeated rebinding and release events whereby a heparin molecule dissociating from a fibronectin molecule will rebind to neighboring fibronectin molecules multiple times before diffusing into the bulk (14). Accordingly, the combined heparin association and dissociation phases should offer sufficient time for heparin to interact with the majority of the immobilized fibronectin layer and be almost completely released prior to VEGF or ECD binding. Interestingly, the presence of the dissociation phase during heparin pretreatment had no effect on VEGF binding. This suggests that the 1-min association phase at that heparin concentration was sufficient to expose all available VEGF-binding sites on the immobilized fibronectin layer. Indeed, the heparin-catalyzed structural rearrangement of fibronectin is very fast and able to reach completion within 1 min (14). At the same time, the fact that VEGF binding remained the same, even in the absence of heparin dissociation, confirms that, in this experimental setup, VEGF interactions with fibronectin-bound heparin were negligible. For ECD, however, heparin dissociation was necessary in order to observe any binding. In the absence of heparin dissociation, we observed a first fast initial burst of binding, but the RU returned quickly to the baseline levels. There was no difference in ECD binding after a 10-min or 30-min heparin dissociation phase. These data suggest that heparin interferes and competes with ECD for binding to fibronectin, possibly because the ECD-binding sites on fibronectin overlap with one of the heparin-binding sites. On the contrary, the heparin presence had no effect on VEGF binding to fibronectin, suggesting that despite the similarities in VEGF and ECD binding to fibronectin, the sites for the two ligands are not identical. It is interesting to notice that the 40 kDa domain of fibronectin, which contains the VEGF-binding sites, can also bind a multitude of other growth factors and cytokines, suggesting the existence of mechanisms that regulate the availability of such a great number of binding sites within a limited domain and orchestrate the binding events (13). If the ECD-binding sites are also located in this domain, the presence of heparin chains (and not only their catalytic action) may be part of such a regulatory mechanism, determining which binding events take place. In future studies utilizing fibronectin fragments and mutants, we plan to map precisely the VEGF and ECD-binding sites on fibronectin.

The model that described best the SPR experimental data revealed the presence of two populations of fibronectin molecules (site A and site B) that can bind VEGF and ECD with different affinities (Fig. 5). Although the two-sites model described the data better than the one-site model, there were still discrepancies between the experimental data and the fitted values, especially for VEGF. Statistical analysis suggests that these discrepancies could be explained by the levels of experimental error (Table 2). However, it is possible that they reflect aspects of the interaction not captured adequately by a simple two-sites model, such as rebinding events as have been reported for one-site models (41, 43, 44). We did not incorporate this term in the fitting model to avoid overparameterization, especially since the system of differential equations describing the two-sites interaction does not have an analytical solution. We are currently developing a mathematical model to describe this phenomenon for two-site interactions, which will be presented in a future study.

**Figure 7:**
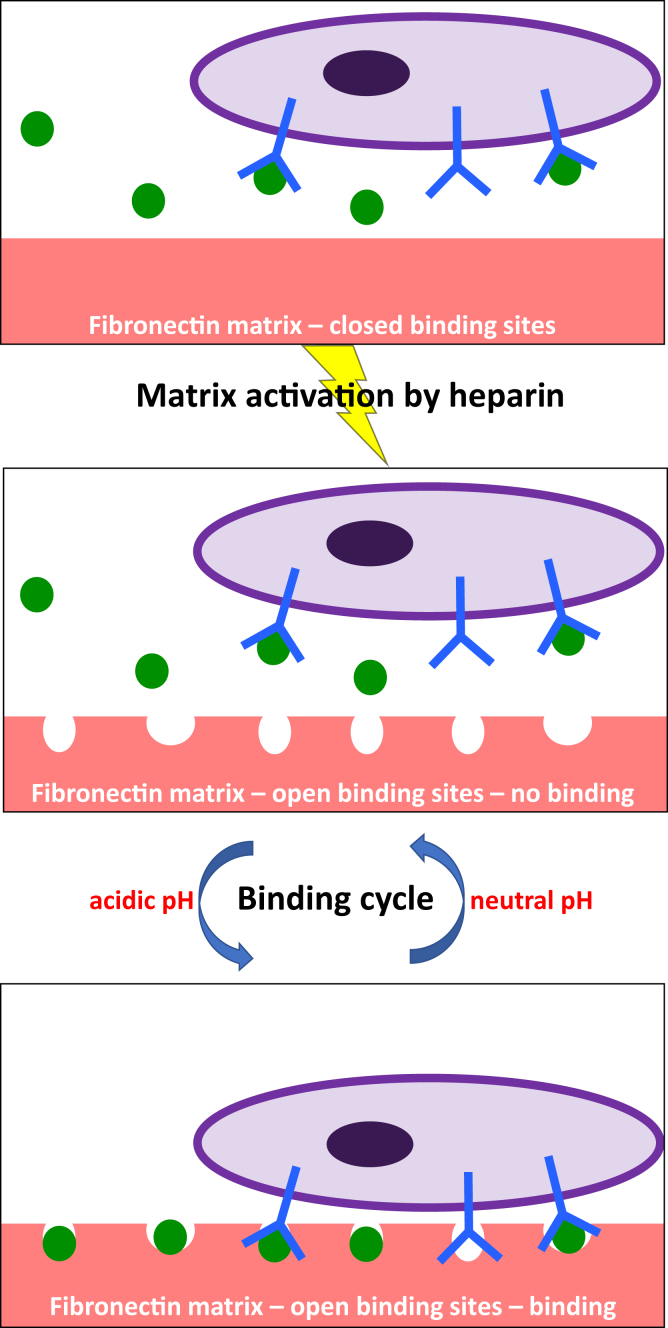
**Schematic representation of interactions between VEGF, VEGFR2, and fibronectin in the extracellular matrix.** Binding of VEGF (*green*) and VEGFR2 (*blue*) at the cell surface (*purple*) to fibronectin within the extracellular matrix (*red*) requires first the action of heparin, which alters the conformation of fibronectin and exposes the binding sites. One set of sites (site A) is characterized by fast association and dissociation rates, leading to a fast turnover (represented by the wider circles in the fibronectin matrix). On the other hand, binding of VEGF and VEGFR2 on the second set of sites (site B) occurs with lower association and dissociation rates, resulting in more stable binding (represented by the narrower circles in the fibronectin matrix). However, binding of either VEGF or VEGFR2 to fibronectin cannot occur unless the pH of the cell microenvironment becomes acidic. When pH returns back to neutral, VEGF and VEGFR2 dissociate from fibronectin, completing the binding cycle.

According to the model, site A, which is the minority of the total population (20–30%), is characterized by high association and dissociation rates, whereas site B (70–80% of the total population) has association and dissociation rates lower than those for site A by one or more orders of magnitude (Table 3). Since the binding of VEGF and ECD to fibronectin was negligible in the absence of heparin treatment, this heterogeneity cannot be explained by the presence of fibronectin molecules on which the binding sites have not yet been opened by heparin. Furthermore, the action of heparin is very fast and even if the time of contact with the immobilized fibronectin was increased, the levels of VEGF and ECD binding remained the same. Therefore, it is also unlikely that the two binding populations reflect an incomplete action of heparin. Instead, these results suggest that not all fibronectin molecules respond equally to the action of heparin. Indeed, previous studies by atomic force microscopy (AFM) have identified structural heterogeneity on single fibronectin molecules, with approximately 70% of the visualized molecules adopting a similar configuration (7). The origin of this heterogeneity is not known, but may be related to the inherent variability in the amino acid sequence of the two fibronectin chains generated by alternative splicing (45) and/or the presence of diverse covalently bound glycans (GlyGen database: P02751–15). Irrespectively of the source of this structural heterogeneity, it is possible that when these molecules respond to the heparin action generate binding site B, whereas the rest will generate site A. According to our model, site A is more accessible than site B for ligand binding, leading to higher association rates, but it can also be freed more quickly (higher dissociation rates). The actual values for the kinetic rates result in site A having higher affinity for VEGF, and site B exhibiting higher affinity for ECD, and since site B represents the majority of binding sites, the overall affinity of ECD for the fibronectin matrix would be higher than that of VEGF, which is consistent with the results of the equilibrium studies. Consequently, high affinity binding of VEGF to fibronectin would be characterized by a high turnover rate, whereas ECD binding would be more stable. Moreover, VEGF binding would occur mostly on a small number of sites, whereas ECD could more readily interact with the majority of fibronectin sites within the extracellular matrix. These differences may have an impact on the dynamics of the interactions between the three molecules and fine-tune their functionalities. A model describing VEGF/VEGFR2/fibronectin interactions occurring *in vivo*, extrapolated from our *in vitro* observations, is shown in Figure 7.

The enhanced binding of VEGF and ECD on fibronectin at acidic pH may have significant consequences for angiogenesis. It is known that hypoxia, signalizing the need for angiogenesis, leads to anaerobic metabolism and consequently, decreases locally the extracellular pH (46). In tumors, which also require angiogenesis to sustain their growth and metastasize, there are additional mechanisms stimulating glycolysis, even under normoxic conditions, contributing to the low levels of extracellular pH. There have been cases of tumors with extracellular pH as low as 5.8 (47). Thus, fibronectin would bind the angiogenic factor VEGF and its receptor primarily in areas requiring active angiogenic signaling. Such sequestration of VEGF within the matrix may increase its bioavailability and help create gradients to guide the growth of new vessels. On the other hand, fibronectin-bound VEGF can alter VEGFR2 signaling (48). It is possible that VEGFR2 binding to fibronectin may alter its residence time on the cell membrane, altering its signaling output. Finally, such interactions may facilitate the formation of higher-order complexes, including integrins and neuropilins, fine-tuning the cell response to an angiogenic stimulus. It is interesting that upon switching the pH back to neutral, both fibronectin-bound VEGF and ECD can be quickly released (Fig. 3). This behavior would act as a regulatory mechanism that can potentially terminate VEGF/VEGFR2 angiogenic signaling when the conditions in the extracellular microenvironment cease to be acidic.

CD spectra of VEGF and ECD acquired at neutral (pH 7.5) and acidic (pH 5.5) pH revealed that the pH alteration did not cause major conformational changes on either molecule (Fig. 4). Therefore, the pH dependency of the interactions might rely completely on pH-sensitive amino acids within the binding sites. Using structure-based algorithms to calculate pKa values of individual amino acids, we could identify several residues, especially His residues, on both VEGF and ECD that could act as pH sensors (Table 1). Control experiments with nontagged VEGF or His-tagged VEGF isoforms that do not interact with fibronectin suggest that the His residues of the tag used for recombinant protein production are not involved in the enhanced binding observed at acidic pH. In ECD, most of the candidate residues were found on domains 3, 4, and 5, and their side chains were not involved in interactions with VEGF in the VEGF-VEGFR2 complex, or in stabilizing the secondary and tertiary structure of the protein. In VEGF, on the other hand, most of the candidate residues were involved in interactions either with VEGFR2 or with the second monomer in the biologically active dimeric form of VEGF, except for one His residue in the C-terminal domain of VEGF (His125), which is free from interactions and could participate in interactions with fibronectin. However, this residue is missing in VEGF_121_, another VEGF isoform that can also bind to fibronectin in a pH-dependent manner (12). Therefore, His125 cannot be the only pH-sensitive residue involved in VEGF–fibronectin interactions. It is known that pKa values are very sensitive to the microenvironment of the residue, and electrostatics, conformational fluctuations, and solvent thermodynamics can cause pKa shifts (49). Moreover, structural information is fragmentary for both VEGF and ECD. High-resolution structures exist only for individual domains and not for the full-length proteins. Therefore, the prediction of the pKa values of individual residues based on the known structures should be interpreted with caution. Nonetheless, His residues often act as pH sensors, modulating protein structure and protein–protein interactions (36, 37).

Decreasing the pH may also affect the binding sites on fibronectin. Several studies have shown that fibronectin undergoes conformational changes in response to pH changes (50). It is possible that these conformational changes also affect the VEGF and ECD-binding sites on fibronectin. We did calculate pKa values on surface-exposed residues in the FNIII 12–14 domains of fibronectin, which contains the VEGF and possibly the ECD-binding sites, based on two alternative conformations that have been reported. Interestingly, we identified a large number of basic (pKa > 10) and acidic (pKa < 4), but none that could act as pH sensors (5.5 < pKa < 7.5). This suggests an elegant mechanism whereby the action of heparin regulates fibronectin conformation to expose VEGF/ECD-binding sites, whereas the local pH regulates ligand binding. However, given the size and flexibility of fibronectin, we cannot exclude the possibility that in the full-length protein different amino acid residues are exposed in the FNIII 12–14 domains or that they have different pKa values. Furthermore, as already discussed, additional regions of fibronectin may be involved in ECD binding.

Understanding VEGF/VEGFR2 interactions with fibronectin within the extracellular matrix may have implications for cancer therapy. Interestingly, some of the His residues in ECD that may be involved in the interactions with fibronectin are found mutated in several cancer cases (unpublished data). Understanding how fibronectin may affect the angiogenic potential of VEGF may help design novel drugs that are more specific, increase their efficacy, and decrease their side effects. Future studies identifying the exact binding sites on all molecular partners, and exploring the consequences of fibronectin binding to the structure and angiogenic signaling of VEGF/VEGFR2 complexes, can open up possibilities for novel therapeutic approaches.

## Experimental procedures

### Materials

The pFastBac vector (cat. no 10360–014) and the CellFectin transfection reagent were purchased from Invitrogen. Ampicillin, kanamycin, chloramphenicol, tetracyclin, and X-Gal were purchased from VWR. Gentamicin and IPTG were purchased from Sigma. The Sf21 TiterHigh AC free cell line (European Collection of Authenticated Cell Culture, cat. no 05030202) was obtained from Sigma. The High Five (BTI-Tn-5B1-4; Hi5) cell line was kindly provided by prof. Ballmer-Hofer at the Paul Scherrer Institute, Switzerland. The insect cell culture medium SF-4 Baculo Express (cat. no 9–00F38-K) was purchased from BioConcept. The 100x Gibco Antibiotic/Antimycotic supplement (containing 10,000 units/ml penicillin, 10,000 μg/ml streptomycin, and 25 μg/ml amphotericin B) was purchased from Thermo Fisher Scientific (cat. no 15240062). The HisTrap Excel, Hi Trap G HP, and S200 10/30 Superdex chromatography columns were from GE Healthcare. Sephadex PD10 MiniTrap G-25 1-ml columns were purchased from Sigma. Glass-bottom 96-well plates were obtained from Life Systems Design (cat. no 324001), and hydrophobic polystyrene F-bottom 96-well plates were from Greiner (cat. no 655 101). The 1-step Turbo TMB substrate was from VWR, and the Clarity ECL chemiluminescence substrate was from BioRad. TGX (4–20% gradient) Stain-Free electrophoresis gels were purchased from BioRad. The Cy3-NHS ester used for protein fluorescence labeling was obtained from Lumiprobe (cat. no 11020). Heparin sodium salt from porcine intestinal mucosa was purchased from Sigma (cat. no H3393). The mouse anti-His (cat. no H1029-2 Ml) and anti-VEGFR2 (V3003-2 Ml) antibodies were purchased from Sigma. The HRP-conjugated goat anti-mouse secondary antibody (from Jackson Immunolabs) was purchased from MILAN Analytica (cat. no 115–035–003). Human plasma fibronectin was obtained from Millipore (cat. no FC010). The recombinant VEGF_165_ protein, the ECD cDNA, and the DH10beta Embac YFP cells were kindly provided by prof. Ballmer-Hofer at the Paul Scherrer Institute, Switzerland. All other reagents were obtained from Sigma.

### Cell culture

The Sf21 *Spodoptera frugipedra* and High Five (Hi5) *Trichoplusia ni* insect cell lines were cultured at 27 °C in the serum-free SF-4 Baculo Express medium, supplemented with 1000 units/ml penicillin, 1000 μg/ml streptomycin, and 0.25 μg/ml amphotericin B. The cultures were maintained in suspension at a density of no more than 2.0 ∗ 10^6^ cells/ml.

### Recombinant protein expression and purification

ECD, encompassing the seven domains of the extracellular part of human VEGFR2 and carrying a 6x His Tag at the C-terminus, was cloned in the pFastBac plasmid. DH10beta Embac YFP electrocompetent bacteria were transformed with the ECD/pFastBac plasmid by electroporation and positive colonies were isolated after ampicillin, kanamycin, chloramphenicol, tetracycline, gentamicin, IPTG, and X-Gal selection for bacmid generation. Sf21 cells grown in 6-well plates at a density of 0.5 ∗ 10^6^ cells/ml were transfected with the ECD bacmid using CellFectin according to the manufacturer’s instructions, and the secreted virus was harvested from the medium after 48 h incubation at 27 °C (V_0_). The virus was amplified by infection of suspension Sf21 cell cultures (3 ml of V_0_ in a 500-ml culture) at a density of 0.5 ∗ 10^6^ cells/ml. The V_1_ viral stock (1.5 ∗ 10^7^ pfu/ml) was harvested 9 days after infection, when 90% of the cells exhibited YFP fluorescence. The virus was amplified a second time by infection of suspension Sf21 cell cultures (10 ml of V_1_ in a 1-L culture) at a density of 1.0 ∗ 10^6^ cells/ml. The V_1_ viral stock (2.2 ∗ 10^8^ pfu/ml) was harvested 4 days after infection, when more than 90% of the cells exhibited YFP fluorescence. Aliquots of the viral stocks were stored at –80 °C.

The V_2_ viral stock was used for protein production. Suspension Hi5 cultures (4 ∗ 1 L) were infected with 10 ml/L of the V_2_ viral stock at a density of 1.0 ∗ 10^6^ cells/ml. The culture was monitored daily for cell viability and infection (by fluorescence). When more than 90% of the cell population exhibited YFP fluorescence (usually 4 days post infection), the culture medium was collected by centrifugation (5000 x *g* for 15 min at 10 °C), filtered, and loaded on 4-ml HisTrap Excel columns. The His-tagged protein was eluted by applying a linear 0–100% buffer B gradient in 20-column volumes (buffer A: 50 mM Tris, 300 mM NaCl, 10 mM imidazole pH 8.0; buffer B: 50 mM Tris, 300 mM NaCl, 500 mM imidazole pH 8.0). The fractions of the peak were collected, dialyzed against 20 mM Tris, pH 8.0, and loaded on an ion exchange 1-ml HiTrap Q HP column. The proteins bound to the column were eluted applying a linear 0–100% buffer B gradient in 20-column volumes (buffer A: 20 mM Tris, pH 8.0; buffer B: 20 mM Tris, 300 mM NaCl, pH 8.0). The fractions corresponding to the ECD (based on molecular weight by SDS-PAGE analysis) were collected, dialyzed against SEC buffer (50 mM Hepes, 150 mM NaCl, 5% glycerol, pH 7.5), concentrated to 0.5 ml, and loaded on an S200 10/30 Superdex size exclusion column. The proteins were eluted with one-column volume SEC buffer and 0.5 ml fractions were collected. The fractions corresponding to monomeric ECD were pooled, concentrated, and stored at –80°C. Protein storage at 4 °C for 15 days led to slight fragmentation. Protein yields ranged from 10 to 30 μg/L of culture. Protein purity during the sequential chromatographic steps was monitored by SDS-PAGE (Fig. S20).

VEGF_165_ containing an N-terminal 6x His tag, expressed in *Pichia Pastoris*, and purified by immobilized metal affinity and size-exclusion chromatography, was kindly provided by prof. Ballmer-Hofer (Paul Scherrer Institute, Switzerland). Human plasma fibronectin was purchased from EMD Millipore. The purity of both VEGF and fibronectin was assessed by SDS-PAGE (Fig. S20).

### ELISA-based binding assay

Fibronectin was added on glass-bottom or hydrophobic polystyrene 96-well plates (20 μg/ml in PBS; 50 μl/well) and was allowed to adsorb overnight at 4 °C. Subsequently, the plate was placed on ice, the solution was aspirated, the adsorbed layer was washed with phosphate-based saline (PBS) (three times; 50 μl/well each time), and was allowed to interact with VEGF or ECD in binding buffer (150 mM NaCl, 25 mM Hepes) at pH 7.5 or 5.5, for 1 h at 4 °C. The solution was then aspirated, the plate was washed with binding buffer (three times; 50 μl/well each time), and the bound ligand was released with the appropriate extraction buffer. After incubation in the extraction buffer (1 min–1 h), the solution was transferred into a second 96-well polystyrene plate. To ensure complete transfer, an additional 50 μl of the extraction buffer was added to the original wells, pipetted twice up and down, and mixed with the material from the first extraction. The extracted ligand was allowed to adsorb on a new plate for 1 h on ice. Then, the solution was aspirated, the plate was washed with assay buffer (three times; 50 μl/well each time), and was incubated with an anti-His or an anti-VEGFR2 primary antibody for 1 h on ice. The solution was aspirated, the plate was washed with assay buffer (three times; 50 μl/well each time), and was incubated with an HRP-labeled secondary antibody for 1 h on ice. The solution was aspirated, the plate was washed with assay buffer (three times; 50 μl/well each time), and the signal was developed accordingly, for absorbance or chemiluminescence measurements. For absorbance, 50 μl of TMB substrate was added on the plate and incubated for 10 min at room temperature. The reaction was stopped by adding 50 μl 1 M H_2_SO_4_ and absorbance was measured at 450 nm and 570 nm using a Microplate reader Infinite 200 PRO (Tecan Group AG, Switzerland). The signal at 570 nm, corresponding to light scattering, was subtracted from the signal at 450 nm, corresponding to TMB absorbance, in order to correct for any features from the well bottom interfering with the measurement. For chemiluminescence, 100 μl of ECL substrate was added on the plate, and the luminescence signal was measured after 10 min, applying 1000 ms integration time and automatic gain using a Microplate reader Infinite 200 PRO. The luminescence signal was corrected by subtracting the sum of the signal from the surrounding eight wells multiplied by the correction factor 0.0454, which was determined by repeated measurements of the blanc in different wells, surrounded by samples of different luminescence values (Fig. S6).

### Determination of fibronectin desorption

Fibronectin was fluorescently labeled with Cy3 according to standard amine chemistry procedures. Briefly, fibronectin (0.5 mg in 0.1 M NaHCO_3_, pH 8.4) was mixed with 20x molar excess of Cy3-NHS dye and was incubated for 1.5 h on ice protected from light. The labeled protein was separated from free dye by size-exclusion column chromatography using 1-ml Sephadex G-25 columns. The protein was eluted in PBS and its concentration and labeling ratio were determined by measuring the absorbance at 280 nm and 550 nm (NanoDrop). Cy3-labeled fibronectin in PBS was added on black polystyrene or glass-bottom 96-well plates and was allowed to adsorb overnight at 4 °C. After the total fluorescence was measured using a Microplate reader Infinite 200 PRO (excitation wavelength = 550 nm; emission wavelength = 595 nm), the solution was aspirated, the adsorbed layer was washed three times with PBS, and then was subjected to a series of incubations (1 h each, on ice) with different buffers (PBS containing 1 mg/ml BSA and 0.05% Tween20 or 5 M NaCl, 25 mM Hepes, pH 7.5). After each incubation, the solution was aspirated, the protein layer was washed three times with PBS, and fluorescence was measured as above to determine how much fibronectin remained adsorbed on the well surface.

### Surface plasmon resonance (SPR) measurements

SPR measurements were performed with a BiaCore X instrument (GE Healthcare) using low-density carboxymethyldextran hydrogel (500 nm) biosensor chips with two flow cells (CDM500 L, Xantec bioanalytics GmbH, Germany). Fibronectin was immobilized on flow cell 2 according to the manufacturer’s instructions, while flow cell 1 was used as a reference. Briefly, the chip surface in both flow cells was activated by injecting 70 μl of a 1:1 mixture of 100 mM NHS and 400 mM N-ethyl-N’(dimethylaminopropyl)carbodiimide hydrochloride (EDC) at a flow rate of 5 μl/min. Within 3 min after the surface activation, 70 μl fibronectin was injected on flow cell 2 only (0.2, 2, or 20 μg/ml in 10 mM sodium acetate buffer, pH 5.0) at a flow rate of 5 μl/min. The remaining free carboxyl groups on the surface of both flow cells were inactivated by injecting 70 μl 1 M ethanolamine, pH 8.0 at a flow rate of 5 μl/min. The interaction between immobilized fibronectin and VEGF or ECD was studied in running buffer (150 mM NaCl, 25 mM Hepes, pH 5.5) that had been autoclaved, filtered, and degassed. Different injection volumes (30 or 60 μl) and flow rates (10, 25, 50, and 100 μl/min) were tested, and the final measurements were performed with 60 μl VEGF or ECD (20 nM, 50 nM, 100 nM, 200 nM, 500 nM, and 1000 nM) at 50 μl/min. For assay optimization, only the association part of the sensogram was recorded. For complete analysis of the interaction and model fitting, the dissociation phase was monitored for a total of 720 s. A pretreatment step of the immobilized fibronectin with 1 μg/ml heparin in running buffer (50 μl at 50 μl/min) was included immediately before the VEGF or ECD injections. After each VEGF or ECD injection, the surface was regenerated with 2 M NaCl and 0.05 N NaOH (50 μl each at 50 ml/min). All measurements were performed at room temperature.

### Kinetic modeling

The SPR data were analyzed with multiple models to extract kinetic parameters for the interactions between fibronectin and VEGF or ECD. Interestingly, we observed a drop in RU values prior to the nominal end of the association phase (59 ± 12 s for VEGF and 65 ± 10 s for ECD instead of 72 s), which, however, showed no correlation with VEGF/ECD concentration (Fig. S19). Although we cannot exclude the possibility that this may reflect some aspect of the binding interactions, we believe that it is caused by experimental error. Strengthening this conclusion is the fact that such time discrepancies were observed for both flow cells and were seemingly random. In the absence of any solid mechanistic justification for ligand dissociation during the association phase, the kinetic parameters reported here have been extracted using variable dissociation time in all cases. Before describing the individual models, we outline the mass conservation equations on which all further considerations are based. We assume that all VEGF or ECD molecules introduced in the system either remain in the flow phase or bind to fibronectin that is immobilized on the surface; as a consequence, neglecting all other possible interactions, the total number of VEGF or ECD molecules is conserved.

The mass conservation equations that we used are derived from a two-compartment model developed previously (51), based on the complete description of the kinetic/mass transfer phenomena in an SPR experiment through partial differential equations (PDE). This model, although simpler than the original PDEs, is able to recapitulate accurately the key parameters describing the time evolution of the system. Briefly, the model takes into consideration two compartments: a flowing bulk phase with a time-invariant concentration of ligands (*C*_*T*_) and a narrow volume slit (*V*_*i*_) in the proximity of the surface (*S*) where receptors have been immobilized at an initial concentration *R*_*T*_ (Fig. 6). Ligand molecule exchange occurs between the flowing bulk phase and *V*_*i*_ (mass transfer), as well as between *V*_*i*_ and *S* (binding/unbinding events). Owing to these phenomena, the ligand concentration in the volume *V*_*i*_ (*C*), as well as the amount of free (*R*) and receptor-bound (*B*) ligand molecules at *S*, varies with time. Applying the principle of mass conservation to the volume *V*_*i*_ and to the surface S, we obtain the following system of equations:

$$\left\{ \begin{array}{l} V_{i}\frac{dC}{dt}={\dot{R}}_{in,V_{i}}-{\dot{R}}_{out,V_{i}}+k_{m}S\left(C_{T}-C\right)\\ S\frac{dB}{dt}={\dot{R}}_{in,S}-{\dot{R}}_{out,S}\\ C\left(t=0\right)=0\\ B\left(t=0\right)=0 \end{array}\right.$$

In the system above, ${\dot{R}}_{in,V_{i}}$ and ${\dot{R}}_{out,V_{i}}$ are, respectively, the rates of ligand molecule gain and loss in the volume *V*_*i*_, as a consequence of binding/unbinding reactions. ${\dot{R}}_{in,S}$ and ${\dot{R}}_{out,S}$ refer to the same quantities with respect to the surface S. $k_{m}$ is the convective mass-transfer coefficient for the exchange of ligand molecules between the flowing bulk and the volume *V*_*i*_.

It is possible to express the quantities ${\dot{R}}_{in,V_{i}}$, ${\dot{R}}_{out,V_{i}}$, ${\dot{R}}_{in,S}$ and ${\dot{R}}_{out,S}$ in a way that is specific for the surface S:

$${\dot{R}}_{in,V_{i}}=S{\hat{R}}_{in,V_{i}}\;;\;{\dot{R}}_{out,V_{i}}=S{\hat{R}}_{out,V_{i}}\;;\;{\dot{R}}_{in,S}=S{\hat{R}}_{in,S}\;;\;{\dot{R}}_{out,S}=S{\hat{R}}_{out,S}$$

The newly defined quantities ${\hat{R}}_{in,V_{i}}$, ${\hat{R}}_{out,V_{i}}$, ${\hat{R}}_{in,S}$ and ${\hat{R}}_{out,S}$ have molar flux units. Consequently, the system of equations transforms as follows:

$$\left\{ \begin{array}{l} V_{i}\frac{dC}{dt}=S\left[{\hat{R}}_{in,V_{i}}-{\hat{R}}_{out,V_{i}}+k_{m}\left(C_{T}-C\right)\right]\\ S\frac{dB}{dt}=S\left[{\hat{R}}_{in,S}-{\hat{R}}_{out,S}\right]\\ C\left(t=0\right)=0\\ B\left(t=0\right)=0 \end{array}\right.$$

It is possible to divide the first equation by *V*_*i*_ and the second by *S*. The ratio between S and *V*_*i*_ is the reciprocal of the characteristic height (*h*_*i*_) of the volume slit, where it is assumed that the mass transfer and binding/unbinding phenomena occur. Accordingly, the system of equation is transformed as follows:

$$\left\{ \begin{array}{l} \frac{dC}{dt}=\frac{1}{h_{i}}\left[{\hat{R}}_{in,V_{i}}-{\hat{R}}_{out,V_{i}}+k_{m}\left(C_{T}-C\right)\right]\\ \frac{dB}{dt}={\hat{R}}_{in,S}-{\hat{R}}_{out,S}\\ C\left(t=0\right)=0\\ B\left(t=0\right)=0 \end{array}\right.$$

In the case of multiple-site reactions, the second expression in the system can be split into multiple equations, each describing the behavior of a specific reaction site.

Below, we present in detail the different models that have been used for fitting the experimental data (Fig. 6). In all models, the term receptor corresponds to the monomeric chain of each fibronectin dimer and the term ligand to VEGF or ECD.

#### One-site model

This is the simplest model, assuming one population of receptor molecules, with each receptor molecule possessing one ligand-binding site. We keep the same nomenclature outlined before for the binding sites of the free (*R*) and ligand-bound (*B*) receptor molecules, and we describe the ligand–receptor interactions using single association ($k_{a}$) and dissociation ($k_{d}$) kinetic constants. Consequently:

$${\hat{R}}_{in,V_{i}}={\hat{R}}_{out,S}=k_{d}B$$

$${\hat{R}}_{in,S}={\hat{R}}_{out,V_{i}}=k_{a}CR=k_{a}C\left(R_{T}-B\right)$$

The system of equation becomes:

$$\left\{ \begin{array}{l} \frac{dC}{dt}=\frac{1}{h_{i}}\left[-k_{a}C\left(R_{T}-B\right)+k_{d}B+k_{m}\left(C_{T}-C\right)\right]\\ \frac{dB}{dt}=+k_{a}C\left(R_{T}-B\right)-k_{d}B\\ C\left(t=0\right)=0\\ B\left(t=0\right)=0 \end{array}\right.$$

#### Two-sites model

This model assumes two populations of receptor molecules with surface concentrations $R_{T,1}=\alpha R_{T}$ and $R_{T,2}=\left(1-\alpha \right)R_{T}$, respectively, with α ranging from 0 to 1. Each receptor molecule possesses a single ligand-binding site, as in the one-site model, but with distinct association ($k_{a,1}$ and $k_{a,2}$) and dissociation ($k_{d,1}$ and $k_{d,2}$) rates. Ligand binding to receptor molecules belonging to either of the two populations (*R*_1_ and *R*_2_) occurs independently, generating molecular species *B*_1_ and *B*_2_. Thus, the second equation of the system that describes the evolution of the ligand-bound receptors is split into two different equations, one for each receptor population. The kinetic terms can be described as follows:

$${\dot{R}}_{in,V_{i}}=k_{d,1}B_{1}+k_{d,2}B_{2}$$

$${\dot{R}}_{out,V_{i}}=k_{a,1}CR_{1}+k_{a,2}CR_{2}=k_{a,1}C\left(R_{T,1}-B_{1}\right)+k_{a,2}C\left(R_{T,2}-B_{2}\right)$$

$${\dot{R}}_{in,S,1}=k_{a,1}CR_{1}=k_{a,1}C\left(R_{T,1}-B_{1}\right)$$

$${\dot{R}}_{in,S,2}=k_{a,2}CR_{2}=k_{a,2}C\left(R_{T,2}-B_{2}\right)$$

$${\dot{R}}_{out,S,1}=k_{d,1}B_{1}$$

$${\dot{R}}_{out,S,2}=k_{d,2}B_{2}$$

Therefore, the system of equations becomes:

$$\left\{ \begin{array}{l} \frac{dC}{dt}=\frac{1}{h_{i}}\left[-k_{a,1}C\left(R_{T,1}-B_{1}\right)-k_{a,2}C\left(R_{T,2}-B_{2}\right)+k_{d,1}B_{1}+k_{d,2}B_{2}+k_{m}\left(C_{T}-C\right)\right]\\ \frac{dB_{1}}{dt}=k_{a,1}C\left(R_{T,1}-B_{1}\right)-k_{d,1}B_{1}\\ \frac{dB_{2}}{dt}=k_{a,2}C\left(R_{T,2}-B_{2}\right)-k_{d,2}B_{2}\\ C\left(t=0\right)=0\\ B_{1}\left(t=0\right)=0\\ B_{2}\left(t=0\right)=0 \end{array}\right.$$

#### Parallel reactions model

This model assumes that each receptor molecule possesses two ligand-binding sites that act independently. From a modeling perspective, this scenario can be treated as a special case of the two-sites model, where $R_{T,1}=R_{T,2}=\;R_{T}/2$ and α = 0.5. Therefore, we used the same system of equations as for the two-sites model under these restrictions.

#### Consecutive reactions model

This model also assumes that each receptor molecule possesses two ligand-binding sites, but in contrast to the parallel reactions model, the binding sites do not act independently; instead, the first binding event is required before the second binding can occur. In this case, we do not consider the concentration of binding sites (*B*_1_ and *B*_2_) as was done in the other cases, but instead, the concentration of the various molecular species of the receptor: free (*R*), bound to the first ligand (*R*_*A*_), and bound to the second ligand (*R*_*2A*_), with $R_{T}=R+\;R_{A}+\;R_{2A}$. We consider the most general case, whereby the two binding events occur with distinct association and dissociation rates: $k_{a,R}$ and $k_{d,R_{A}}$ for the first reaction (*R* to *R*_A_), and $k_{a,R_{A}}$ and $k_{d,R_{2A}}$ for the second (R_*A*_ to *R*_*2A*_). As for the two-sites model, the second equation of the system has to be split in multiple equations to describe the behavior of each molecular species. Then, the kinetic terms can be computed as follows:

$${\dot{R}}_{in,V_{i}}=k_{d,R_{A}}R_{A}+k_{d,R_{2A}}R_{2A}$$

$${\dot{R}}_{out,V_{i}}=k_{a,R}CR+k_{a,R_{A}}CR_{A}$$

$${\dot{R}}_{in,S,R}=k_{d,R_{A}}R_{A}$$

$${\dot{R}}_{out,S,R}=k_{a,R}CR$$

$${\dot{R}}_{in,S,R_{A}}=k_{a,R}CR+k_{d,R_{2A}}R_{2A}$$

$${\dot{R}}_{out,S,R_{A}}=k_{a,R_{A}}CR_{A}+k_{d,R_{A}}R_{A}$$

$${\dot{R}}_{in,S,R_{2A}}=k_{a,R_{A}}CR_{A}$$

$${\dot{R}}_{out,S,R_{2A}}=k_{d,R_{2A}}R_{2A}$$

Accordingly, the final system of equations becomes:

$$\left\{ \begin{array}{l} \frac{dC}{dt}=\frac{1}{h_{i}}\left[k_{d,R_{A}}R_{A}+k_{d,R_{2A}}R_{2A}-k_{a,R}CR-k_{a,R_{A}}CR_{A}+k_{m}\left(C_{T}-C\right)\right]\\ \frac{dR}{dt}=-k_{a,R}CR+k_{d,R_{A}}R_{A}\\ \frac{dR_{A}}{dt}=k_{a,R}CR+k_{d,R_{2A}}R_{2A}-k_{a,R_{A}}CR_{A}-k_{d,R_{A}}R_{A}\\ \frac{dR_{2A}}{dt}=k_{a,R_{A}}CR_{A}-k_{d,R_{2A}}R_{2A}\\ C\left(t=0\right)=0\\ R\left(t=0\right)=R_{T}\\ R_{A}\left(t=0\right)=0\\ R_{2A}\left(t=0\right)=0 \end{array}\right.$$

#### Solution of the system of ordinary differential equations (ODEs) and optimization of the parameter values

The free parameters to be determined for each model are shown in Table 4. For the parameter *h*_*i*_, we used a fixed value of 10^−5^ *m*, which is a reasonable approximation based on the characteristic size of the SPR microfluidics chip. Moreover, results from a previous study (51) show that the solutions of the equations describing an SPR experiment are insensitive to the value of *h*_*i*_, which was also true here (data not shown). The values of the free parameters were determined by fitting the experimental data. To avoid overfitting, the experimental curves collected at different values of *C*_*T*_ were fitted simultaneously (global fitting). During the association phase, the values of *C*_*T*_ were considered equal to the experimental ones, whereas at the beginning of the dissociation phase was set to zero. The units of the experimental data (RU) were converted to $mol/m^{2}$, based on previously reported assumptions (51), using the following equation:

L(RU)=L(molm2)⋅10−4(m2cm2)⋅MW(gmol)⋅1010RUgcm2

**Table 4:** List of parameters to be estimated by experimental data fitting

Model	Free parameters
One-site model	$k_{a},\;k_{d},\;k_{m},\;R_{T}\;$
Two-sites model	$k_{a,1},k_{a,2},\;k_{d,1},\;k_{d,2},\;k_{m},\;R_{T},\;\alpha \;$
Parallel reactions model	$k_{a,1},k_{a,2},\;k_{d,1},\;k_{d,2},\;k_{m},\;R_{T}$
Consecutive reactions model	$k_{a,R},\;k_{a,R_{A}},k_{d,R_{A}},k_{d,R_{2A}},k_{m},\;R_{T}\;$

According to the models presented on Fig. 6, describing the interactions between fibronectin and VEGF or ECD, the listed parameters should be extracted from fitting the SPR dose responses to each of the proposed models.

MW represents the molecular weight of the ligand and is equal to 45 or 83 g/mol for VEGF or ECD, respectively. *L* represents the total ligand surface concentration and is calculated for the different models as follows:

L=B(One−site model)

$$L=B_{1}+B_{2}\;\left(\text{Two}-\text{sites model};\text{ Parallel reactions model}\right)$$

$$L=R_{A}+2R_{2A}\;\left(\text{Consecutive reactions model}\right)$$

The ordinary differential equations were discretized and solved using the explicit Euler method, with a time interval of $dt=\;10^{-2}\;s$. Given an initial set of parameter estimates, the ordinary differential equations system was solved and the output was compared with the experimental results. Unconstrained fitting was performed in Matlab using the function *lsqcurvefit*, which minimizes the following quantity:

$$F=\underset{C_{T}}{\sum }\underset{t}{\sum }{\left(L_{exp}\left(C_{T},t\right)-L_{mod}\left(C_{T},t\right)\right)}^{2}$$

$L_{exp}$ and $L_{mod}$ represent the total ligand surface concentration as derived from the experimental data or the fitted model, respectively. The summation is performed over the time points of a single data set (inner sum) and over different data sets collected at different values of $C_{T\;}$ (outer sum).

The solution of the system can be performed according to two different scenarios. The first scenario consists in considering mass transfer limitations. In such a case, the systems of equations are identical with the ones explained above. The second scenario assumes mass transfer not to be limiting (rapid mixing): ligand exchange between the flowing bulk and the volume $V_{i}$ is faster that the characteristic binding/unbinding timescales. Under such an assumption, $C=C_{T}$ replaces the first equation in all the systems discussed in the previous paragraphs.

### Statistical analysis of curve fitting

The experimental data were fitted with all four models (one-site, two-sites, parallel reactions, and consecutive reactions model), under conditions of rapid mixing or mass transfer. The beginning of the dissociation phase was set to the maximum RU value achieved during the association phase (variable dissociation time). To ensure as much as possible that the solution did not represent a local minimum, the following strategy was adopted for all cases: the fitting was repeated multiple times, with sets of initial values for the free parameters that differed by several orders of magnitude. Considering all curves in the data set (with different $C_{T\;}$ values), the residual sum of squares (RMS) was calculated for each solution as the sum of the squared residuals (difference between experimental and fitted value), and the solution with the lowest RMS value was selected. Confidence intervals were then calculated for each of the estimated parameters based on the residual values and the Jacobian matrix, using the *nlparci* Matlab function. Solutions for which the confidence intervals for one or more of the parameters were as large as to include negative values were rejected, indicating overfitting, and that the corresponding model could not be used to fit the data. For each model that gave a solution with well-defined confidence intervals for all estimated free parameters (Table S2), the following equations were used to calculate various goodness-of-fit indices, where Δ denotes the value of the residuals, n the number of experimental points, and p the number of model parameters:

1. Residual sum of squares: $RMS=\;\sum {\text{Δ}}^{2}$
2. Residual variance: $\sigma^{2}=\;\frac{\sum {\text{Δ}}^{2}}{n-2}$
3. Akaike’s Information Criterion: $AIC=n\cdot log\left(\sigma^{2}\right)+2\cdot p\cdot \frac{n}{n-p-1}$

To assess the fitting, the absolute and relative values of these indices were examined (Table 2). Traditionally, the residual variance (corresponding to what is often termed as Chi square in the SPR literature) is used to assess the goodness of fit in SPR experiments. However, it is difficult to define absolute cutoff values, primarily because the residual variance depends on the level of the average signal. Usually, the fit can be considered acceptable when the square root of the residual variance is comparable to the level of experimental noise (52). To estimate the levels of experimental noise, the dose–response experiments were repeated three times using the same chip and injecting the different VEGF/ECD concentrations in a random order to ensure that the data were free of any systematic errors. The patterns were very similar in replicate experiments, but the absolute values were associated with an experimental error of 6–39% (corresponding to 3–24 RU) for ECD and 15–49% (corresponding to 7–18 RU) for VEGF (Fig. S18). This level of experimental error, significantly higher than that of the baseline fluctuations (4%), might have originated from pipetting errors during dilution and injection, as well as stochastic events during binding in both flow cells. Considering this level of noise, all fits, despite their discrepancies with the experimental data, could be acceptable based on the residual variance values. However, not all fits could be considered equivalent. According to information theory, the Akaike’s information criterion (AIC) can be used for model selection among different models that can fit a certain data set, combining absolute fit with model parsimony (53). In other words, it penalizes for the addition of parameters to the model in order to improve absolute fit. The model with the lower AIC is considered the best describing the data in question (53). According to this criterion, the models generating the best overall fit for the data were the consecutive reactions model for VEGF and the two-sites model for ECD. However, the two-sites model generated curves that resembled more closely the shape of the experimental curves for VEGF (Fig. S15); therefore, the two-sites model was also chosen to describe VEGF binding to fibronectin, even though, according to AIC, it was the second best model regarding overall fit.

### Circular dichroism spectroscopy

CD spectra of VEGF, ECD, and complexes between VEGF and ECD were acquired with a J-815 spectrometer (JASCO) with a quartz cuvette and with a path length of 10 mm. Data were collected at 4 °C in the 250–205 nm wavelength range with a 0.2 nm data pitch, standard sensitivity (100 mdeg), 1 s digital integration time (D.I.T.), 1 nm bandwidth, and 20 nm/min scanning speed under a continuous scanning mode. The average spectrum of five sequential measurements (accumulation = 5) was used for processing. The baseline of each buffer was acquired using the same measurement parameters and was subtracted from each spectrum. The data were converted from mdeg to mean residue ellipticity using the molecular weight of each protein or protein complex: 45 kDa for VEGF, 83 kDa for ECD, and 211 kDa for the ECD/VEGF complex. CD measurements were performed in 150 mM NaCl, 25 mM Hepes, pH 7.5 or 5.5 (binding buffer) or in 10 mM sodium phosphate buffer, pH 7.4 or 5.8 (low ionic strength buffer).

### Bioinformatics analysis

Structure predictions of pKa values of individual amino acid residues at the surface of VEGF, ECD, and the FNIII 12–14 domains of fibronectin were performed with the DelPhiPKa web server, using the known high-resolution structures of VEGF, ECD, and FNIII 12–14 fragments (pdb codes: 2VPF and 2VGH for VEGF, 3V2A, 2X1W, 2X1X, 3S35, 3S36, 3S37, 5OYJ, and 3KVQ for ECD, and 1FNH and 3R8Q for the FNIII 12–14 fibronectin domains). The parameters used for the prediction were those that gave the best results on a benchmark study against an extensive database of experimentally determined pKa values: σ = 0.70, ε_ref_ = 8, ε_ext_ = 80. Three different force fields were tested: AMBER, CHARMM, and PARSE, yielding very similar results (data not shown). The pKa values reported were calculated using the AMBER force field. According to the calculated pKa values, titration simulations were performed to calculate the probability of ionization of each residue at pH 7.5 and 5.5 assuming two microstates: protonated and deprotonated.

## Data availability

All data used for this study are presented in this article in the form of graphs or tables. Raw data, as well as the Matlab source code used for fitting the various models on the SPR data, will be available upon request to the corresponding author: Maria Mitsi, maria.mitsi@alumni.ethz.ch, current affiliation: Ectica Technologies, Switzerland.

## Supporting information

This article contains supporting information.

## Conflict of interest

The authors declare that they have no conflicts of interest with the contents of this article.
