# Supplementary material for: VEGF and VEGFR2 bind to similar pH-sensitive sites on fibronectin, exposed by heparin-mediated conformational changes
Source: J Biol Chem. 2021 Mar 24;296:100584. doi: 10.1016/j.jbc.2021.100584 (PMC8102423; doi:10.1016/j.jbc.2021.100584)
Supplement: Figures and Tables [file mmc1.pdf]

Supplementary Information

VEGF and VEGFR2 bind to similar pH-sensitive sites on fibronectin, exposed by heparin-mediated conformational changes

Mattia Usuelli[1], Timmy Meyer[1], Raffaele Mezzenga[1#], Maria Mitsi[1*]

[1]Laboratory of Food and Soft Materials, Department of Health Sciences and Technology, ETH Zurich, Zurich, Switzerland

[*]Corresponding author: Maria Mitsi
E-mail: maria.mitsi@alumni.ethz.ch

[#]Co-corresponding author: Raffaele Mezzenga
E-mail: raffaele.mezzenga@hest.ethz.ch

Running title: VEGFR2 binding to fibronectin

I. Development and optimization of an ELISA-based binding assay (Figures S1-S6; Table S1)

Initially, we attempted to detect the fibronectin-bound ligand directly, using an appropriate primary antibody and an HRP-labeled secondary antibody. We found that the addition of both BSA (1 mg/ml) and Tween20 (0.05%) was necessary and sufficient to suppress the non-specific binding of the antibodies on the plate surface and the adsorbed protein layers (Fig. S1). However, successive incubations with these blocking agents led to significant fibronectin desorption from the polystyrene plate surface (Fig. S2). On the contrary, fibronectin adsorption was much more stable on glass plates, where successive incubations with the BSA and Tween20 containing buffer had no effect (Fig. S2). Thus, we performed the binding assays on glass plates.

To recapitulate earlier observations (ref. 7 in the main text), VEGF binding was tested at two different pH values (pH 7.5 and 5.5) with or without pre-treatment of the adsorbed fibronectin with heparin. However, the non-specific binding of VEGF on the plate surface was very high under all conditions. The amount of adsorbed VEGF on the naked glass substrate was almost the same as that on fibronectin or a number of blocking proteins, including bovine serum albumin (BSA), egg-white albumin, and beta-lactoglobulin (Fig. S3). High concentrations of gelatin reduced VEGF non-specific binding by approximately 50% but this was still not sufficient to uncover the specific VEGF-fibronectin interactions, especially when higher VEGF concentrations were tested (Fig. S3). We attempted to reduce VEGF adsorption on non-specific binding sites by adding blocking proteins (BSA or milk) and detergents (Tween20) in the buffer during binding. The combination of milk and Tween20 worked very well at neutral pH (pH 7.5), but at acidic pH (pH 5.5) had a limited effect (Fig. S4). In a final attempt, we combined the blocking properties of gelatin as a substrate and milk as an additive in the binding buffer. We performed the assay on fibronectin adsorbed on a gelatin gel (several mm in thickness) polymerized inside the plate wells using 20% gelatin in water. However, the gelatin gel itself gave high background values, resulting in negative results and very large errors, preventing its further use (data not shown).

Since we were unable to reduce the non-specific binding of VEGF on the plate, we followed an indirect approach in order to detect the specifically-bound VEGF to surface-immobilized fibronectin. According to a previous study (ref. 7 in the main text), incubation with a high ionic strength solution (5 M NaCl)

could release fibronectin-bound VEGF. Thus, we added to the assay an additional extraction step with 5 M NaCl, followed by re-adsorption of the released VEGF on a second plate and detection by ELISA. Since in this assay format the adsorbed fibronectin does not come in contact with the ELISA blocking buffers containing BSA and Tween20, which would lead to its desorption from polystyrene plates, there was no further need to use glass plates for the assay. Instead, we used hydrophobic polystyrene plates, on which, as reported earlier (ref. 7 in the main text), adsorbed fibronectin adopts a compact conformation, and is amenable to structural regulation by heparin. We confirmed that the 5 M NaCl extraction did not lead to any fibronectin desorption from the polystyrene plate (Fig. S5). Additionally, we switched the detection method to luminescence, which exhibited a larger linear range for VEGF detection (Fig. S6).

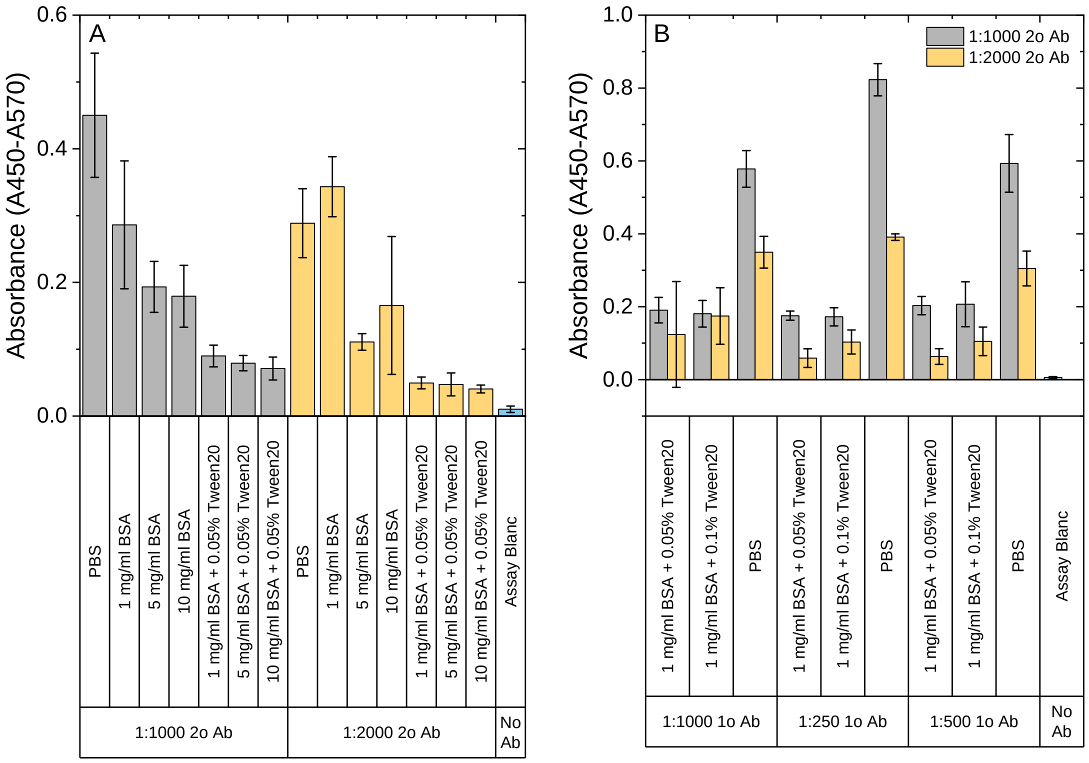

Figure S1. The combination of BSA and Tween20 is necessary and sufficient to suppress antibody non-specific binding. Fibronectin (20 µg/ml; 50 µl/well) was adsorbed on glass-bottom 96-well plates overnight at 4 °C, and was incubated for 1 h with the HRP-labeled anti-mouse secondary antibody (2o Ab) alone (A) or after a 1-h incubation with the anti-His mouse primary antibody (1o Ab) (B). The antibodies were diluted in PBS without any blocking agent or in PBS containing BSA (1, 5, or 10 mg/ml) in the absence or presence of Tween20 (0.05%). The dilution ratios tested were 1:1000 (gray bars) and 1:2000 (ochre bars) for the secondary antibody, and 1:250, 1:500, and 1:1000 for the primary antibody. Quadruplicate samples were measured, and the data are presented as mean values +/- standard deviation. The blanc value of the assay (adsorbed fibronectin with no antibody added) is also shown for comparison (light blue bar). The addition of Tween20 significantly decreased the non-specific binding of the secondary antibody at both dilutions tested, and had a more pronounced effect than increasing the amount of BSA, even by 10 fold (A). Furthermore, the combination of 1 mg/ml BSA and 0.05% Tween20 was able to suppress the non-specific binding of the primary antibody as well, even at the lowest dilution tested (B). Consequently, all ELISA assays were performed in this buffer (1 mg/ml BSA + 0.05% Tween20 in PBS).

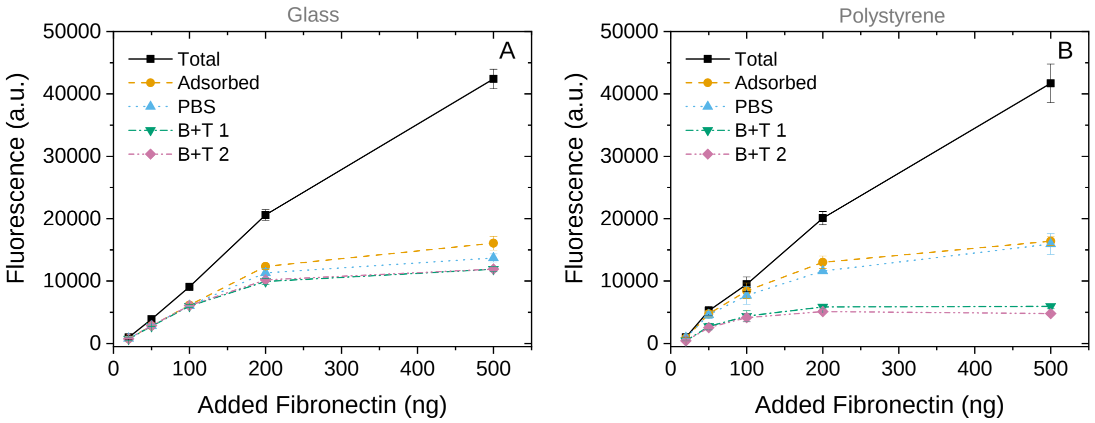

Figure S2. The ELISA blocking buffer containing 1 mg/ml BSA and 0.05% Tween20 (B + T) leads to fibronectin desorption from polystyrene but not from glass. Cy3-labeled fibronectin (20 - 500 ng) was adsorbed on glass-bottom (50 µl/well; A) or half-area black polystyrene (25 µl/well; B) 96-well plates overnight at 4 °C. Fluorescence was measured with a microplate reader (excitation wavelength = 550 nm; emission wavelength = 595 nm) following adsorption (total; black squares), after aspirating the solution and washing the protein layer 3 times with PBS (adsorbed; ochre circles), after 1-h incubation with PBS (PBS, light blue triangles), after 1 h incubation with the ELISA blocking buffer (B + T 1; green inverted triangles), and after a second 1-h incubation with the ELISA blocking buffer (B + T 2; pink rhombuses). Samples were measured in triplicate, and the data are presented as mean values +/- standard deviation.

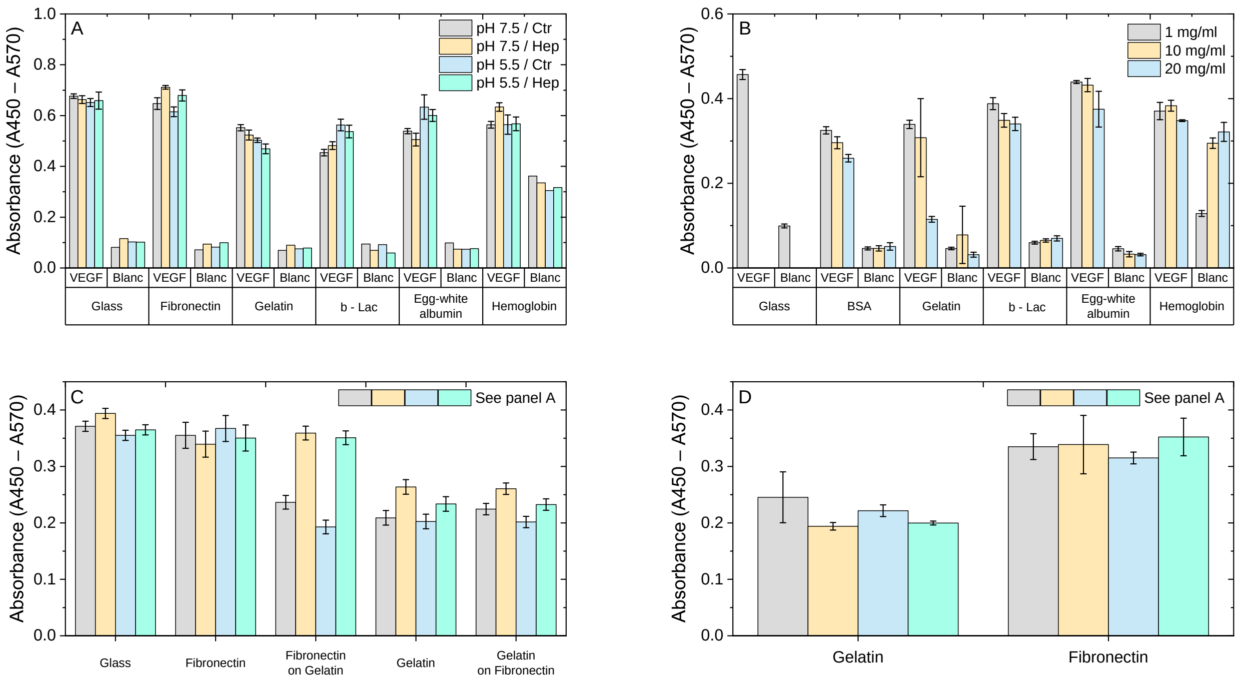

Figure S3. Adsorption of blocking proteins on the assay plate does not reduce non-specific VEGF binding. (A) Fibronectin (20 µg/ml; 50 µl/well) or a series of blocking proteins (1 mg/ml; 50 µl/well) were adsorbed on glass-bottom 96-well plates overnight at 4 °C. Gelatin, beta-lactoglobulin (β-Lac), egg-white albumin, and hemoglobin were tested. The plate was incubated with VEGF (5 µg/ml) in binding buffer (150 mM NaCl, 25 mM Hepes) at pH 7.5 (grey and ochre bars) or 5.5 (light blue and light green bars), in the presence (ochre and light green bars) or absence (grey and light blue bars) of pre-treatment of the adsorbed proteins with 100 µg/ml heparin in PBS. Bound VEGF was detected by ELISA with an anti-His primary antibody (1:1000) and an HRP-labeled secondary antibody (1:2000). Both antibody incubations were performed in ELISA blocking buffer (1 mg/ml BSA + 0.05% Tween20 in PBS). Samples were measured in triplicate, and the data are presented as mean values +/- standard deviation. VEGF adsorption on naked glass, as well as the blanc value of the assay (no antibody added) for each adsorbed protein are shown for comparison. (B) Increasing concentrations (1 mg/ml, grey bars; 10 mg/ml, ochre bars; and 20 mg/ml, light blue bars) of all the blocking proteins used in (A) plus bovine serum albumin (BSA) were adsorbed on glass-bottom 96-well plates (50 µl/well) overnight at 4 °C. VEGF binding was performed as in (A) but only at pH 7.5 in the absence of heparin treatment. Bound VEGF was detected by ELISA as in (A). Samples were measured in triplicate, and the data are presented as mean values +/- standard deviation. VEGF adsorption on naked glass, as well as the blanc value of the assay (no antibody added) for each adsorbed protein are shown for comparison. (C) Fibronectin (20 µg/ml; 50 µl/well) or gelatin (20 mg/ml; 50 µl/well) were adsorbed on glass-bottom 96-well pates overnight at 4 °C (conditions labeled as 'Fibronectin' and 'Gelatin', respectively). In a second step, aimed at testing the ability of gelatin to reduce non-specific binding in the presence of fibronectin without blocking the specific binding sites, fibronectin (20 µg/ml; 50 µl/well) was added on top of the adsorbed gelatin (condition labeled as 'Fibronectin on Gelatin'), and gelatin (20 mg/ml; 50 µl/well) was added on top of the adsorbed fibronectin (condition labeled as 'Gelatin on Fibronectin'). The proteins were allowed to adsorb for 1 h on ice. The plate was incubated with VEGF (5 µg/ml) in binding buffer (150 mM NaCl, 25 mM Hepes) at pH 7.5 or 5.5, in the presence or absence of pre-treatment of the adsorbed proteins with 100 µg/ml heparin in PBS. The color code used in the same as in (A). Bound VEGF was detected by ELISA as in (A). Samples were measured in triplicate, and the data are presented as mean values +/- standard deviation. The data presented have been corrected by subtracting from the raw data the value of the blanc (no added antibodies), for each corresponding condition. (D) Same as in (C) but with 10 µg/ml VEGF and including conditions

‘Fibronectin’ and ‘Gelatin’ only, without the second protein adsorption step. The color code used is the same as in (A).

The data presented here demonstrate the high levels of non-specific VEGF binding on the assay substrate and the failure of a series of blocking proteins to reduce it to levels comparable to the assay blanc values. The only promising protein to block VEGF non-specific binding was gelatin, especially when used at high concentrations (20 mg/ml). However, the blocking effects of gelatin varied between experiments, and were not as pronounced when higher VEGF concentrations (10 μg/ml) were tested.

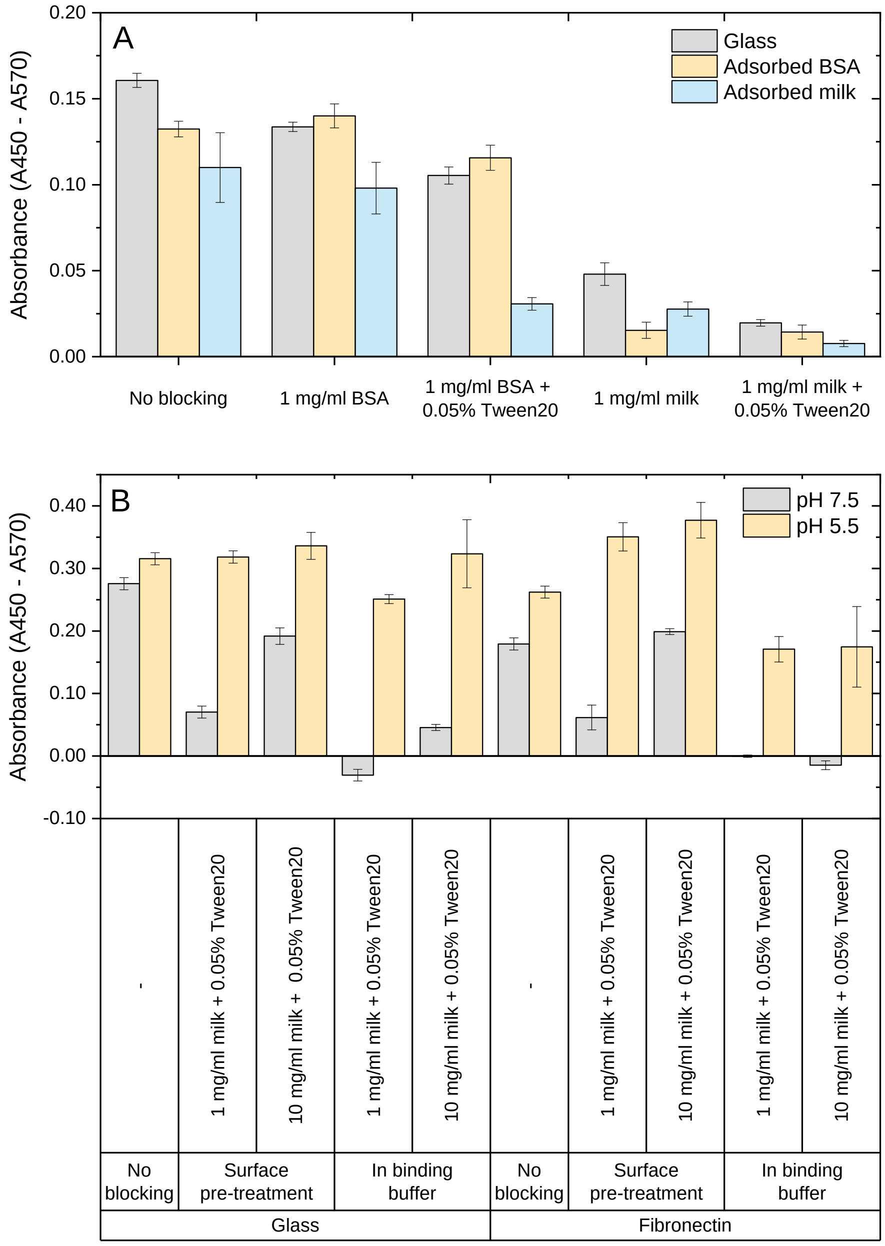

Figure S4. Milk reduces the non-specific binding of VEGF at pH 7.5 but not at pH 5.5. (A) BSA or milk (2 mg/ml; 50 µl/well) were adsorbed on glass-bottom 96-well plates for 1 h on ice. The surface (naked glass, grey bars; adsorbed BSA, ochre bars; adsorbed milk, light blue bars) was incubated for 1 h with binding buffer (150 mM NaCl, 25 mM Hepes, pH 7.5) in the absence (no blocking) or presence of 1 mg/ml BSA +/- 0.05% Tween20 or 1 mg/ml milk +/- 0.05% Tween20 as blocking agents. The plate was then incubated with VEGF (0.5 µg/ml) in the corresponding buffer for 1 h on ice, and bound VEGF was detected by ELISA with an anti-His primary antibody (1:1000) and an HRP-labeled secondary antibody (1:2000). Both antibody incubations were performed in ELISA blocking buffer (1 mg/ml BSA + 0.05% Tween20 in PBS). Samples were measured in triplicate, and the data are presented as mean values +/- standard deviation. The data presented have been corrected by subtracting from the raw data the value of the blanc (no added antibodies), for each corresponding condition. The data demonstrate the great effect of 1 mg/ml milk + 0.05% Tween20 at pH 7.5 to reduce VEGF non-specific binding to near blanc values, when added to the binding buffer. (B) Fibronectin (2 µg/ml; 50 µl/well) was adsorbed on glass-bottom 96-well plates overnight at 4 °C. The effect of 1 mg/ml milk + 0.05% Tween20 or 10

mg/ml milk + 0.05% Tween20 to suppress VEGF non-specific binding was tested at both pH 7.5 (grey bars) and pH 5.5 (ochre bars), by either surface pre-treatment prior to VEGF binding or by adding the blocking agents during VEGF binding. VEGF binding was performed with 0.5 μg/ml VEGF in binding buffer (150 mM NaCl, 25 mM Hepes, pH 7.5 or 5.5) in the absence (no blocking and in the conditions of surface pre-treatment) or presence of the blocking agents. Bound VEGF was detected by ELISA as in (A). Samples were measured in triplicate, and the data are presented as mean values +/- standard deviation. The data presented have been corrected by subtracting from the raw data the value of the blanc (no added antibodies), for each corresponding condition. Although the presence of milk and Tween20 during VEGF binding at pH 7.5 completely eliminated non-specific binding, its effect at pH 5.5 was limited.

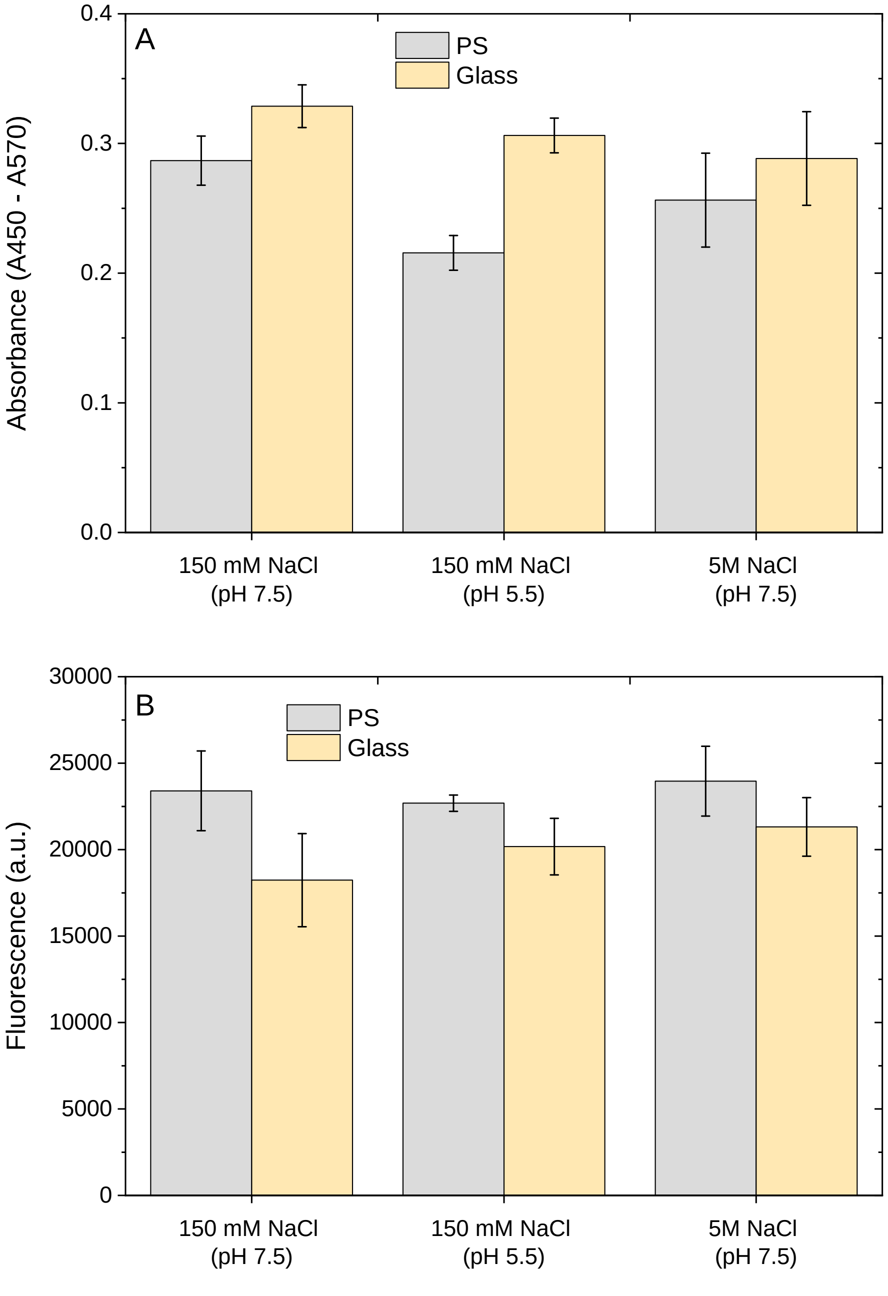

Figure S5. Buffers of different ionic strength and pH do not affect VEGF adsorption to or fibronectin desorption from glass and polystyrene. (A) VEGF was diluted in 3 different buffers (150 mM NaCl, 25 mM Hepes, pH 7.5; 150 mM NaCl, 25 mM Hepes, pH 5.5; 5 M NaCl, 25 mM Hepes, pH 7.5) at a final concentration of 0.5 µg/ml and was adsorbed on polystyrene (grey bars) or glass-bottom (ochre bars) 96-well plates for 1 h on ice (50 µl/well). Adsorbed VEGF was detected by ELISA with an anti-His primary antibody (1:1000) and an HRP-labeled secondary antibody (1:2000), with both antibody incubations performed in ELISA blocking buffer (1 mg/ml BSA + 0.05% Tween20 in PBS). Samples were measured in triplicate, and the data are presented as mean values +/- standard deviation. The data presented have been corrected by subtracting from the raw data the value of the blanc (no added antibodies), for each corresponding condition. The amount of VEGF adsorbed to the different substrates from the different buffers was very similar, with only a small decrease observed in VEGF adsorption to polystyrene at acidic pH. (B) Cy3-labeled fibronectin in PBS was adsorbed on half-area black polystyrene (25 ng; 25 µl/well; grey bars) or glass-bottom (50 ng; 50 µl/well; ochre bars) 96-well

plates overnight at 4 °C. The solution was aspirated, and the protein layer washed 3 times with PBS and treated with the corresponding buffer (150 mM NaCl, 25 mM Hepes, pH 7.5; 150 mM NaCl, 25 mM Hepes, pH 5.5; 5 M NaCl, 25 mM Hepes, pH 7.5) for 1 h on ice. The solution was aspirated, the plate was washed 3 times with PBS, and the amount of remaining protein was determined by fluorescence measurements with a microplate reader (excitation wavelength = 550 nm; emission wavelength = 595 nm). Samples were measured in triplicate, and the data are presented as mean values +/- standard deviation. The values for fibronectin desorption caused by the different buffers were similar. Since the binding buffers containing 150 mM NaCl have a similar ionic strength to that of PBS, which does not cause fibronectin desorption according to the data presented in Fig. S2, we can conclude that incubation with the solution of high ionic strength (5 M NaCl) did not lead to significant fibronectin desorption.

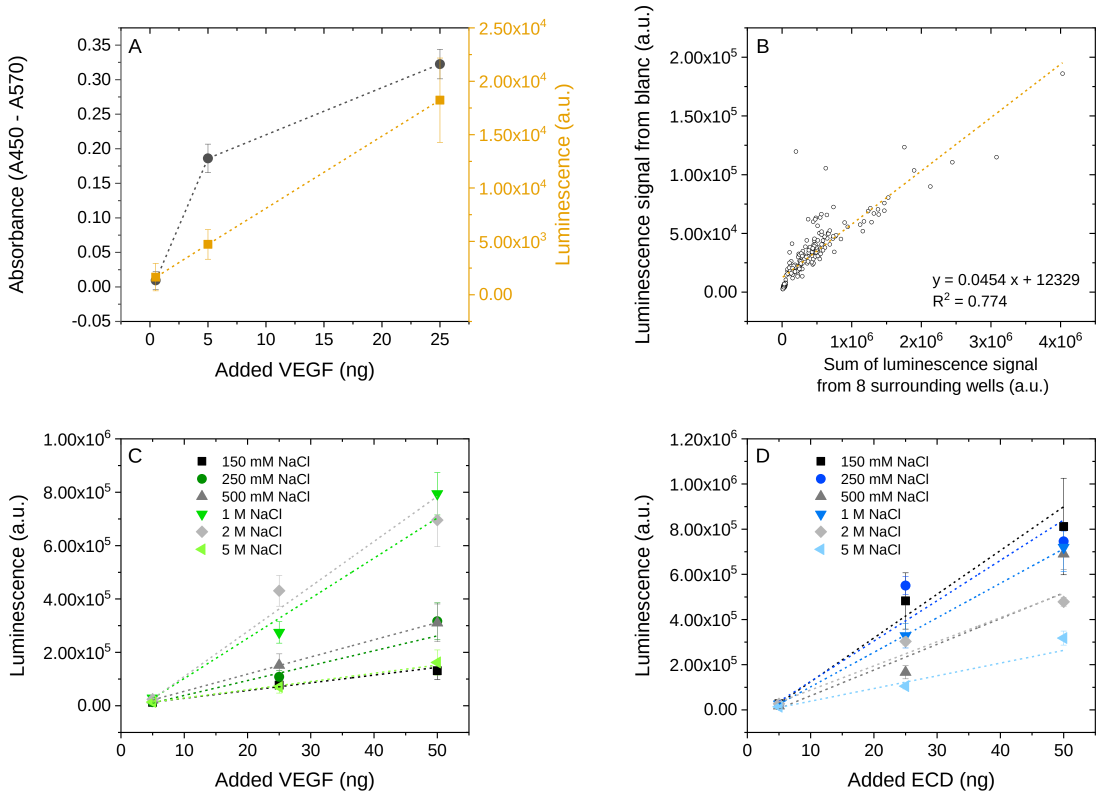

Figure S6. Detection of surface-bound VEGF by luminescence. (A) VEGF (0.5, 5, and 25 ng) was diluted in extraction buffer (5 M NaCl, 25 mM Hepes, pH 7.5) and adsorbed on polystyrene 96-well plates (50 µl/well) for 1 h on ice. Following solution aspiration and 3 washes with PBS, bound VEGF was detected by ELISA with an anti-His primary antibody (1:1000) and an HRP-labeled secondary antibody (1:2000). The signal was developed accordingly and either absorbance at 450 nm (grey circles) or chemiluminescence (ochre squares) was measured with a microplate reader. Samples were measured in triplicate (absorbance) or quadruplicate (luminescence), and the data are presented as mean values +/- standard deviation. The data presented have been corrected by subtracting from the raw data the value of the blanc (no added antibodies), for each corresponding condition. These results demonstrate the superior linear range of the luminescence-based detection method. Consequently, this technique was employed for all further experiments. (B) Since luminescence is emitted in all directions, we sought to determine the effect of the luminescence signal from neighboring wells on each measurement by comparing blanc values (no added antibody) measured in different wells surrounded by samples of varying luminescence intensity. A total of 219 blanc measurements from 7 independent experiments were collected, which show a strong positive linear correlation with the sum of luminescence signal from the 8 surrounding wells, with a slope of 0.0454 and an intercept of 12329 (R^2 = 0.7744). Accordingly, all luminescence measurements were corrected by subtracting the sum of the signal from the surrounding 8 wells multiplied by the correction factor 0.0454. (C, D) Increasing amounts (5, 25, and 50 ng) of VEGF (C) or ECD (D) were diluted in different buffers (150 mM NaCl, 25 mM Hepes, pH 7.5; 250 mM NaCl, 25 mM Hepes, pH 7.5; 500 mM NaCl, 25 mM Hepes, pH 7.5; 1 M NaCl, 150 mM Hepes, pH 7.5; 2 M NaCl, 25 mM Hepes, pH 7.5; 5 M NaCl, 25 mM Hepes, pH 7.5; color code explained in the figure) and adsorbed on polystyrene 96-well plates (50 µl/well) for 1 h on ice. Following solution aspiration and 3 washes with PBS, bound VEGF was detected by ELISA with an anti-His primary antibody (1:1000) and an HRP-labeled secondary antibody (1:2000). Both antibody incubations were performed in ELISA blocking buffer (1 mg/ml BSA + 0.05% Tween20 in PBS). Samples were measured in quadruplicate, and the data are presented as mean values +/- standard deviation. The blanc values (no added antibodies) for each corresponding condition have been subtracted from

the data presented. It is evident that the amount of VEGF/ECD adsorbed and/or detected by the anti-His antibody depends on the ionic strength of the solution. Accordingly, each data series was fit by a line and the slope was used to correct measurements performed in buffers of different ionic strength. The correction factors are shown in Table S1.

II. Equilibrium VEGF/ECD binding to surface-immobilized fibronectin (Figures S7-S8)

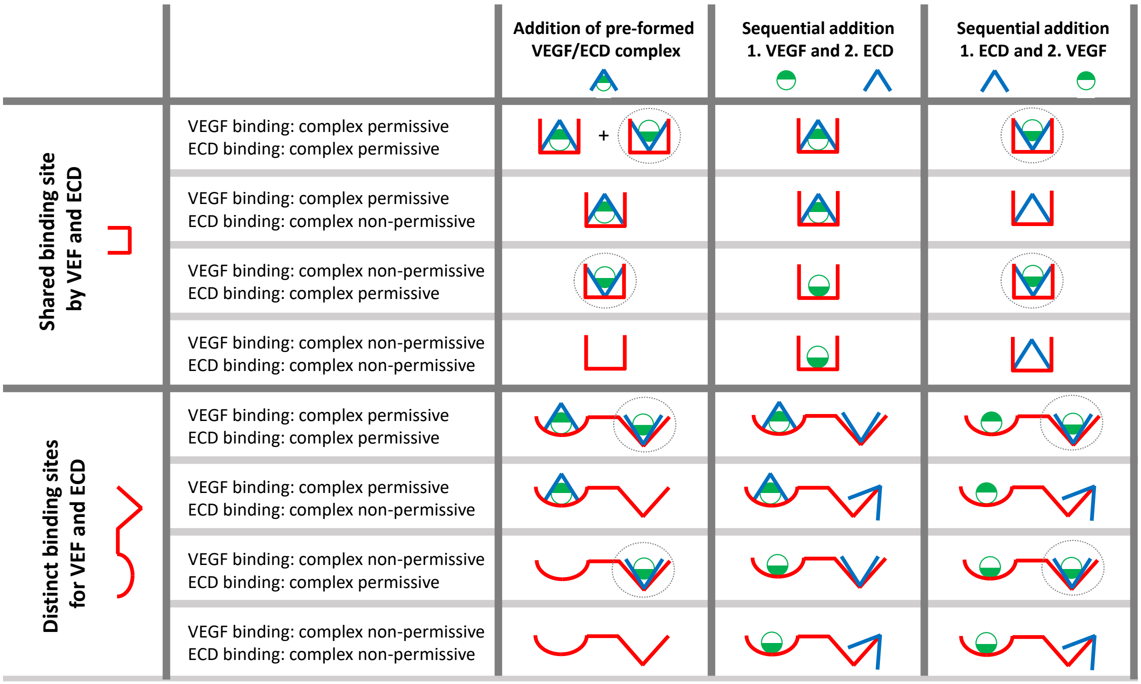

Figure S7. Prediction of molecular species following different VEGF and ECD binding scenarios to fibronectin. We distinguish between shared (red square) and distinct (red circle and triangle) binding sites for VEGF (green circle) and ECD (blue triangle) on fibronectin, which allow or not the formation of a VEGF/ECD complex after binding to fibronectin (complex permissive or non-permissive, respectively). Consequently, for each type of binding sites, we predict which molecular species would be generated by adding the preformed VEGF/ECD complex, VEGF followed by ECD, or ECD followed by VEGF. The complex permissive nature of the VEGF binding sites is depicted schematically by the filled part of the circle representing VEGF projecting away from the fibronectin binding site and being available to bind ECD. On the contrary, when the VEGF binding site of fibronectin is complex non-permissive, the filled part of the circle faces the fibronectin site and cannot interact with ECD. Similarly, the triangle representing ECD is positioned in such a way as to allow interactions with VEGF in the case of complex permissive binding sites, or to block VEGF binding in the case of complex non-permissive interactions. In the molecular species enclosed by the dashed circle (fibronectin-ECD-VEGF, complex permissive), ECD adopts a configuration that cannot be recognized by the anti-VEGFR2 antibody.

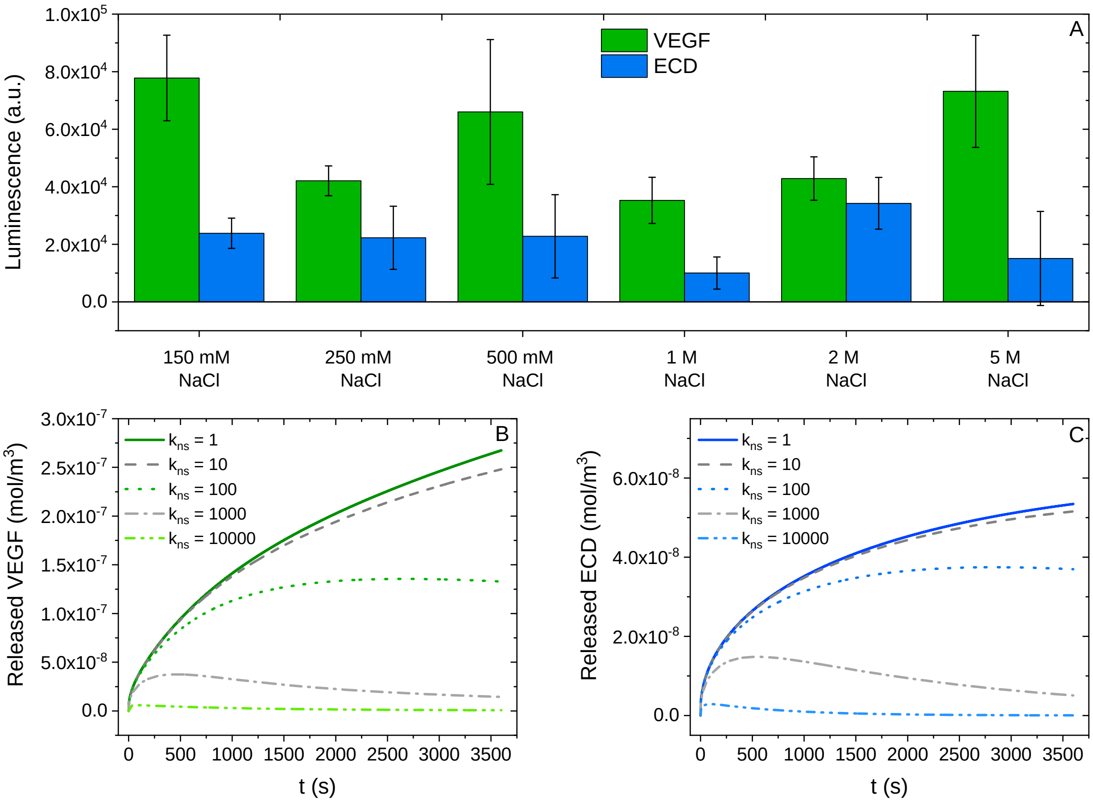

Figure S8. Change of pH is sufficient to release fibronectin-bound VEGF and ECD, but re-adsorption occurs over time. (A) Fibronectin was adsorbed on 96-well polystyrene plates (20 µg/ml; 50 µl/well) overnight at 4 °C. The plate was incubated for 1 h on ice with 50 µg/ml of VEGF (green bars) or ECD (blue bars) in binding buffer (150 mM NaCl, 25 mM Hepes) at pH 5.5 after pre-treatment of the adsorbed fibronectin with 100 µg/ml heparin in PBS (1 h on ice). Bound VEGF and ECD were extracted for 1 h on ice with buffers of increasing ionic strength: 150 mM NaCl, 25 mM Hepes, pH 7.5; 250 mM NaCl, 25 mM Hepes, pH 7.5; 500 mM NaCl, 25 mM Hepes, pH 7.5; 1 M NaCl, 25 mM Hepes, pH 7.5; 2 M NaCl, 25 mM Hepes, pH 7.5; 5 M NaCl, 25 mM Hepes, pH 7.5. The released ligand was re-adsorbed on a second plate, and detected by ELISA with an anti-His primary antibody (1:1000) and an HRP-labeled secondary antibody (1:2000). Both antibody incubations were performed in ELISA blocking buffer (1 mg/ml BSA + 0.05% Tween20 in PBS). Samples were measured in quadruplicate, and the data are presented as mean values +/- standard deviation. The data were corrected for differences in VEGF/ECD adsorption and detection from buffers of different ionic strength (Table S1). (B, C) Simulation of release experiments of VEGF (B) or ECD (C) from fibronectin using a two-sites model with the kinetic parameters shown in Table 3 and assuming an equal number of non-specific and specific (fibronectin) binding sites. Four different association rates for binding to the non-specific sites (k_{ns}) were considered: 1 (solid lines), 10 (dash lines), 100 (dot lines), 1000 (dash-dot lines) and 10000 (dash-double dot lines) $m^3mol^{-1}s^{-1}$. For a given ratio of non-specific/specific binding sites, there exist a k_{ns} threshold that leads to a peak followed by a decrease in ligand release. The more abundant the non-specific binding sites in relation to the fibronectin binding sites, the lower the k_{ns} threshold.

III. Structural determinants of VEGF/ECD-fibronectin interactions (Figure S9)

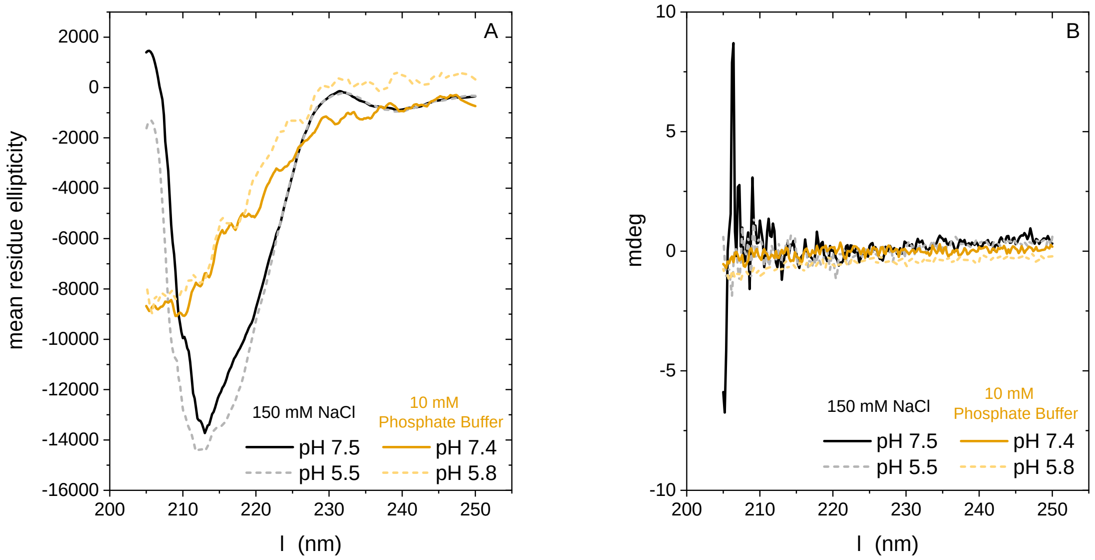

Figure S9. VEGF unfolding at low ionic strength. (A) VEGF was diluted in binding buffer (150 mM NaCl, 25 mM Hepes) at pH = 7.5 (black solid line) or pH = 5.5 (grey dashed line) at a concentration of 0.3 mg/ml or in 10 mM sodium phosphate buffer at pH 7.4 (ochre solid line) or pH = 5.8 (ochre dashed line) at a concentration of 0.05 mg/ml and CD spectra were acquired in the 250-205 nm wavelength range. The data are presented in units of mean residue ellipticity, following smoothing with a Savitzky-Golay filter with a 20-nm window. The data demonstrate that independent of pH, low ionic strength affects the β-sheet dominated structure of VEGF, shifting the negative peak at 215 nm to lower wavelengths, indicative of protein unfolding. Therefore, all CD measurements were performed in buffers containing 150 mM NaCl. (B) Buffer baselines (in mdeg) were collected using the same acquisition parameters as for the VEGF samples shown in (A). The same color code as in (A) applies. The data show that in the presence of 150 mM NaCl, measurements below 210 nm are associated with greater uncertainty, especially at pH 7.5.

IV. Optimization of SPR assay to study and model the kinetics of VEGF/ECD-fibronectin interactions (Figures S10-S19; Table S2)

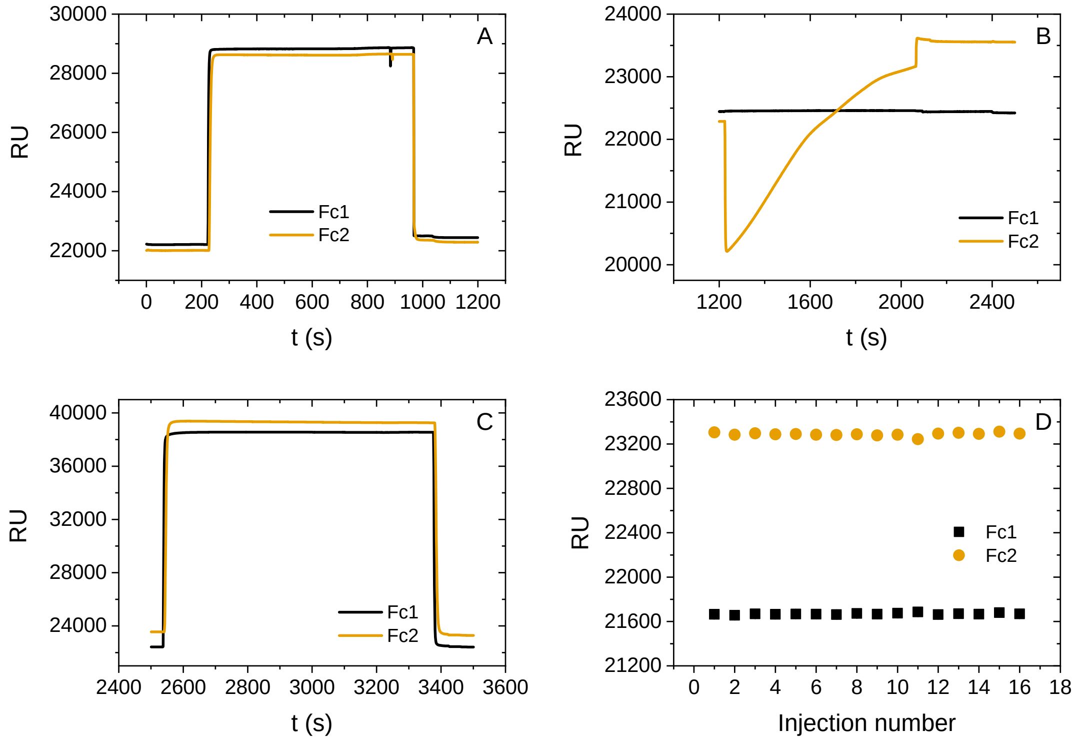

Figure S10. SPR chip functionalization. Fibronectin was immobilized on carboxymethyldextran hydrogel SPR chips based on standard amine chemistry. The first step was surface activation by the addition of NHS and EDC (A), followed by fibronectin (2 µg/ml) immobilization (B), and finally, surface quenching by ethanolamine (C). All steps were performed at a flow rate of 5 µl/min with an injection volume of 70 µl. Surface activation and quenching were performed in both flow cell 1 (Fc1; reference; black) and flow cell 2 (Fc2; sample; ochre), whereas fibronectin immobilization was performed only in flow cell 2. The difference in response units (RU) between flow cells 1 and 2 due to the mass of the immobilized fibronectin was very stable, and persisted after 16 sequential injections of different ligands, followed by 2 M NaCl/0.05 N NaOH regeneration cycles (D). Each point represents an average value from a part of the sensogram encompassing 50-100 values, and is associated with a standard deviation value less than or equal to 4% of the mean.

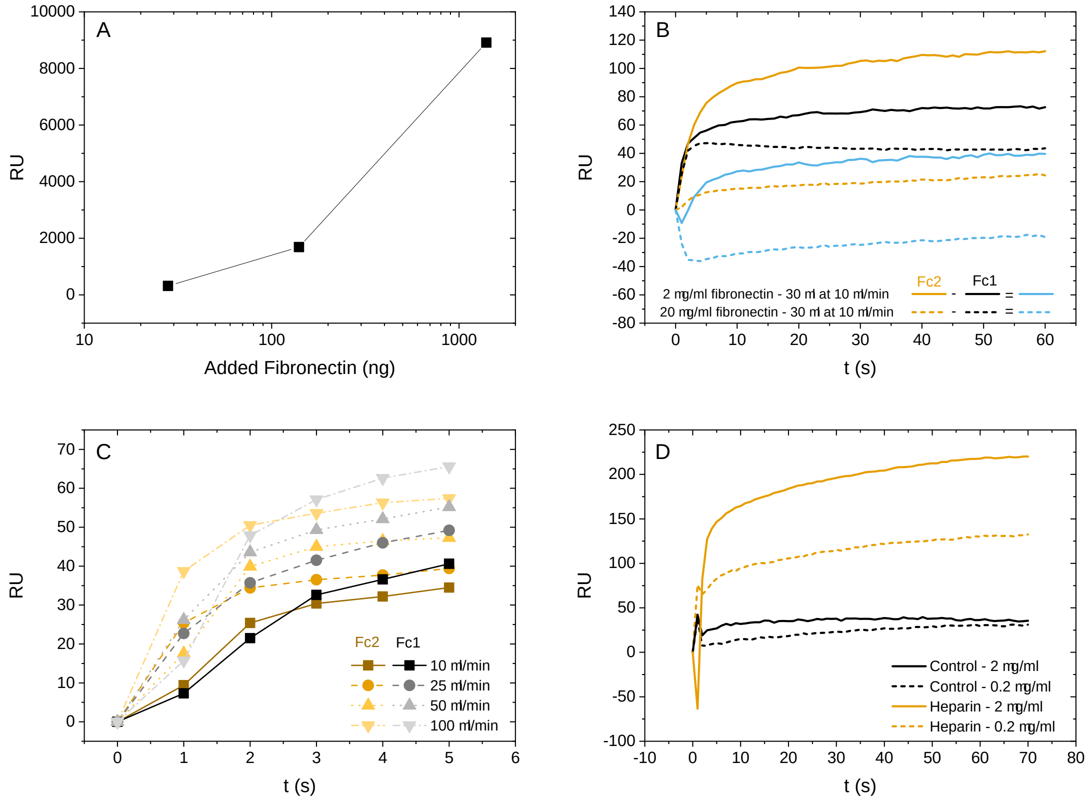

Figure S11. Optimization of fibronectin density and flow rates for SPR measurements. (A) Fibronectin was immobilized on the biosensor chip on flow cell 2 (Fc2) at 3 different densities (2 * 70 µl * 0.2 µg/ml = 28 ng; 70 µl * 2 µg/ml = 140 ng; 70 µl * 20 µg/ml = 1400 ng). Only the difference in RU between sample and reference flow cells (Fc2 – Fc1) is shown. Each point represents an average value from a part of the sensogram encompassing 50-100 values, and is associated with a standard deviation value less than or equal to 4% of the mean. (B) VEGF (30 µl of 1000 nM) was injected at a flow rate of 10 µl/min on chips with 140 ng (solid lines) or 1400 ng (dashed lines) immobilized fibronectin. Black lines correspond to the reference cell Fc1 and ochre lines to the sample cell Fc2. The difference signal (Fc2-Fc1), representing the specific binding events to fibronectin, is shown with the light blue lines. It is evident from the data that VEGF binding to fibronectin is minimal and does not increase by increasing the fibronectin density. On the contrary, at the highest fibronectin density, the value of Fc2 becomes lower than that of Fc1 (possibly because the higher surface coverage by fibronectin reduces VEGF non-specific binding), leading to negative difference curves. (C) Using the chip with the intermediate fibronectin density (140 ng), we tested VEGF binding at different flow rates: 10 µl/min (squares), 25 µl/min (circles), 50 µl/min (triangles), and 100 µl/min (inverted triangles). Only the first 5 s of association are shown for Fc1 (shades of black) and Fc2 (shades of ochre), demonstrating a tendency for faster association as the flow rate increases, which indicates the presence of mass transfer effects. Accordingly, subsequent measurements were performed at a flow rate of 50 µl/min. (D) VEGF (60 µl of 1000 nM) was injected at a flow rate of 50 µl/min on chips with 140 ng (solid lines) or 28 ng (dashed lines) immobilized fibronectin. Only the difference in RU between sample and reference flow cells (Fc2 – Fc1) is shown. VEGF association with fibronectin was recorded in the absence (black lines) or presence (ochre lines) of a pre-treatment step of the immobilized fibronectin with heparin (50 µl of 1 µg/ml at a flow rate of 50 µl/min). Heparin pre-treatment of the immobilized fibronectin significantly increased VEGF binding, and the effect was greater for the higher fibronectin density. Therefore, we chose to use this fibronectin density (70 µl * 2 µg/ml = 140 ng) for further experiments.

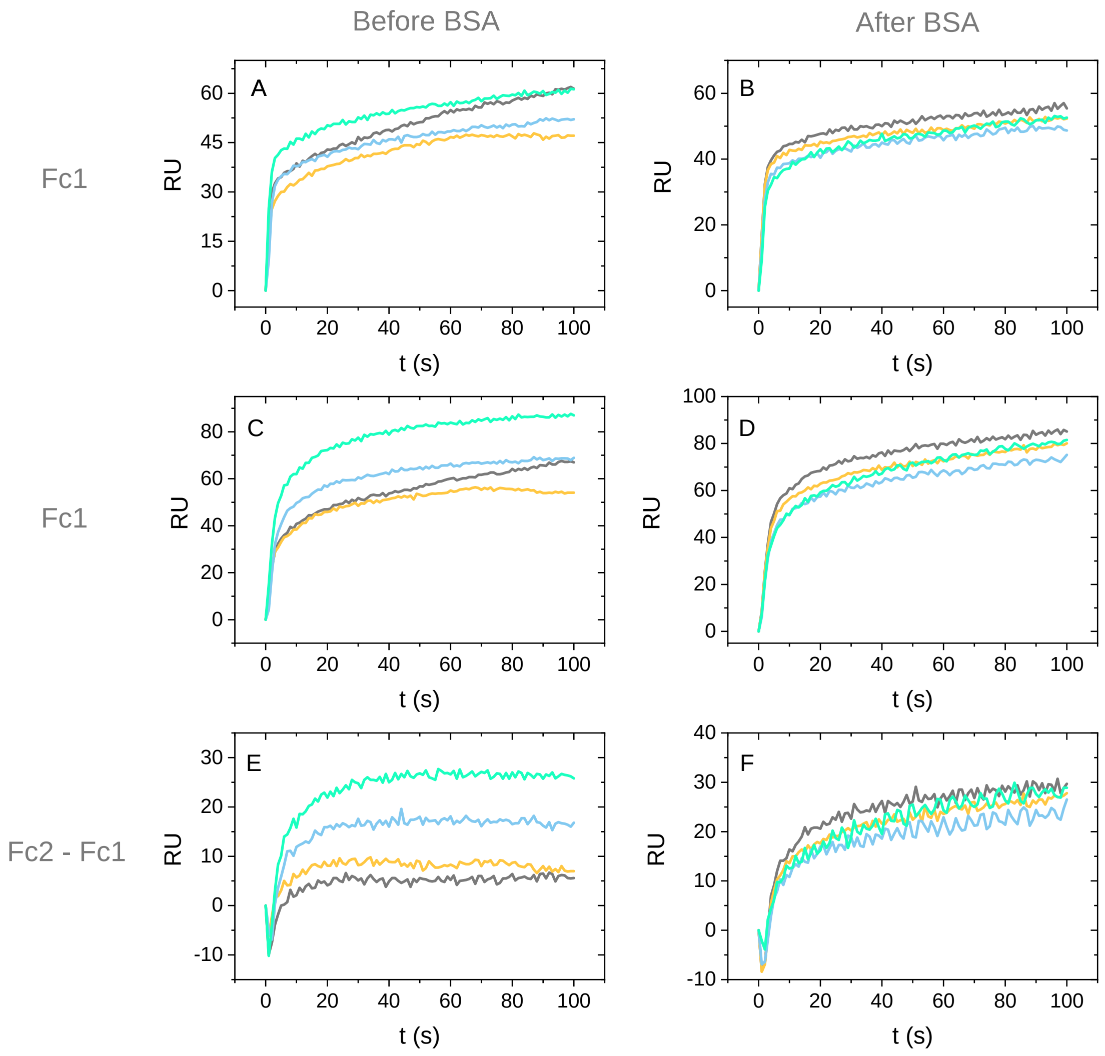

Figure S12. Stabilization of VEGF background binding to fibronectin by BSA injection. VEGF (60 µl of 1000 nM) was injected at a flow rate of 50 µl/min on a chip with 2 µg/ml immobilized fibronectin (140 ng) in the absence of any heparin pre-treatment step (control binding). Fc1 (A, B), Fc2 (C, D) and Fc2-Fc1 (E, F) signals are shown for four sequential injections (first injection: gray lines; second injection: ochre lines; third injection: light blue lines; fourth injection: green lines) performed before (A, C, E) and after (B, D, F) an injection with BSA (60 µl of 1 mg/ml) at a flow rate of 50 µl/min. Before BSA injection, the signal due to VEGF binding increased after each injection, an effect due to the higher values originating from the Fc2 cell. Following BSA injection, this trend disappeared, and the signal stabilized.

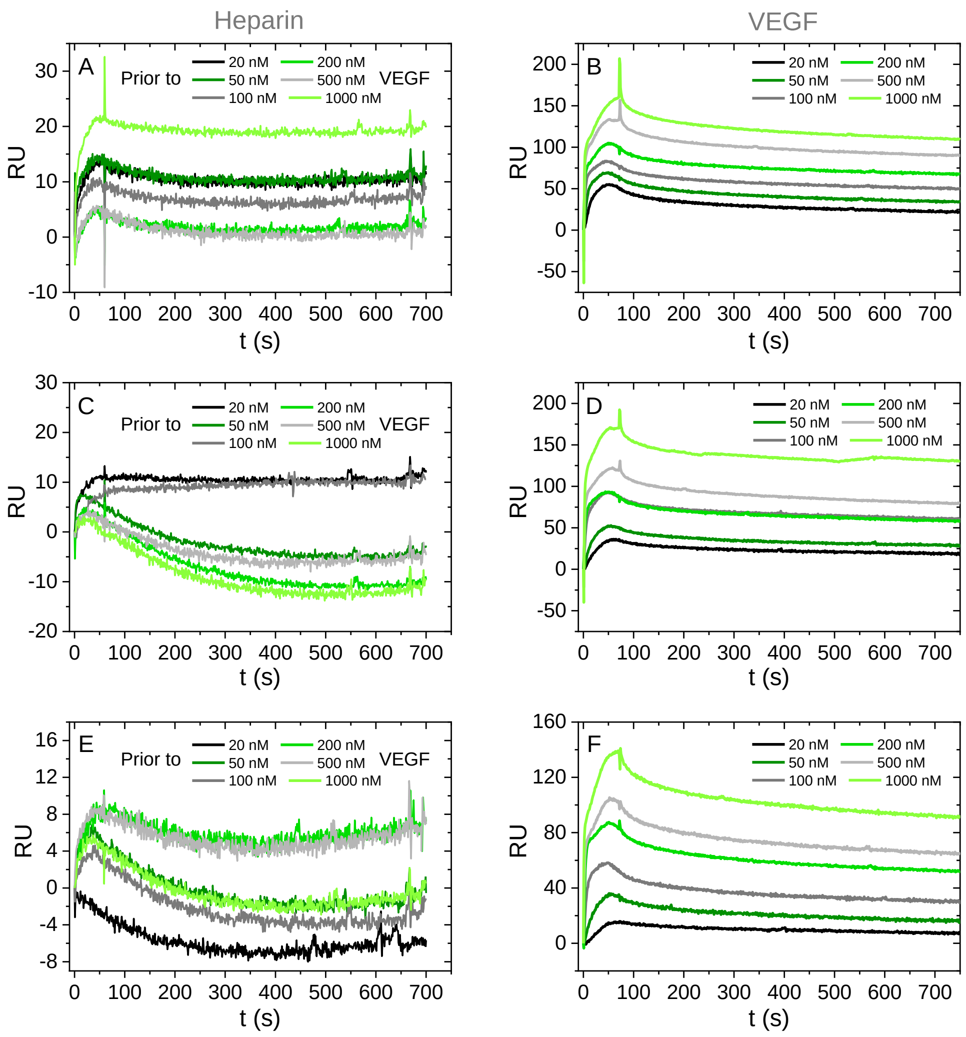

Figure S13. Heparin pre-treatment of immobilized fibronectin increases VEGF binding. Heparin (50 µl of 1 µg/ml) was injected at a flow rate of 50 µl/min on a chip with 2 µg/ml immobilized fibronectin (140 ng). Following association, heparin was allowed to dissociate for 10 min, and immediately afterwards, VEGF was injected (60 µl at 50 µl/min) at different concentrations: 20 nM, 50 nM, 100 nM, 200 nM, 500 nM, and 1000 nM (color code explained in the figure). Following association, VEGF was allowed to dissociate for 720 s. The different VEGF concentrations were injected following a random order in order to ensure that the measurements were free of systematic errors, and the progressive increase in VEGF binding with increasing concentration reflected the true binding. The surface was regenerated with 2 M NaCl and 0.05 N NaOH after every heparin/VEGF binding cycle. The heparin injections are shown in panels (A), (C), and (E), and the corresponding VEGF injections in panels (B), (D), and (F). The dose-response experiment was repeated three times using the same chip (run 1: (A), (B); run 2: (C), (D); run3: (E), (F)). Only Fc2-Fc1 traces are shown. Binding of heparin to fibronectin at this concentration did not result in a significant SPR signal (less than 25 RU for all injections). Occasionally, the sensogram line was not stable and drifted to negative values during the long dissociation phase following the heparin treatment. However, there was no correlation between the RU values following heparin treatment and VEGF binding. Mean and standard deviation values were calculated for VEGF binding from the triplicate experiments. The error associated with each data point was 15-49% (standard deviation normalized against the mean; see Fig. S18).

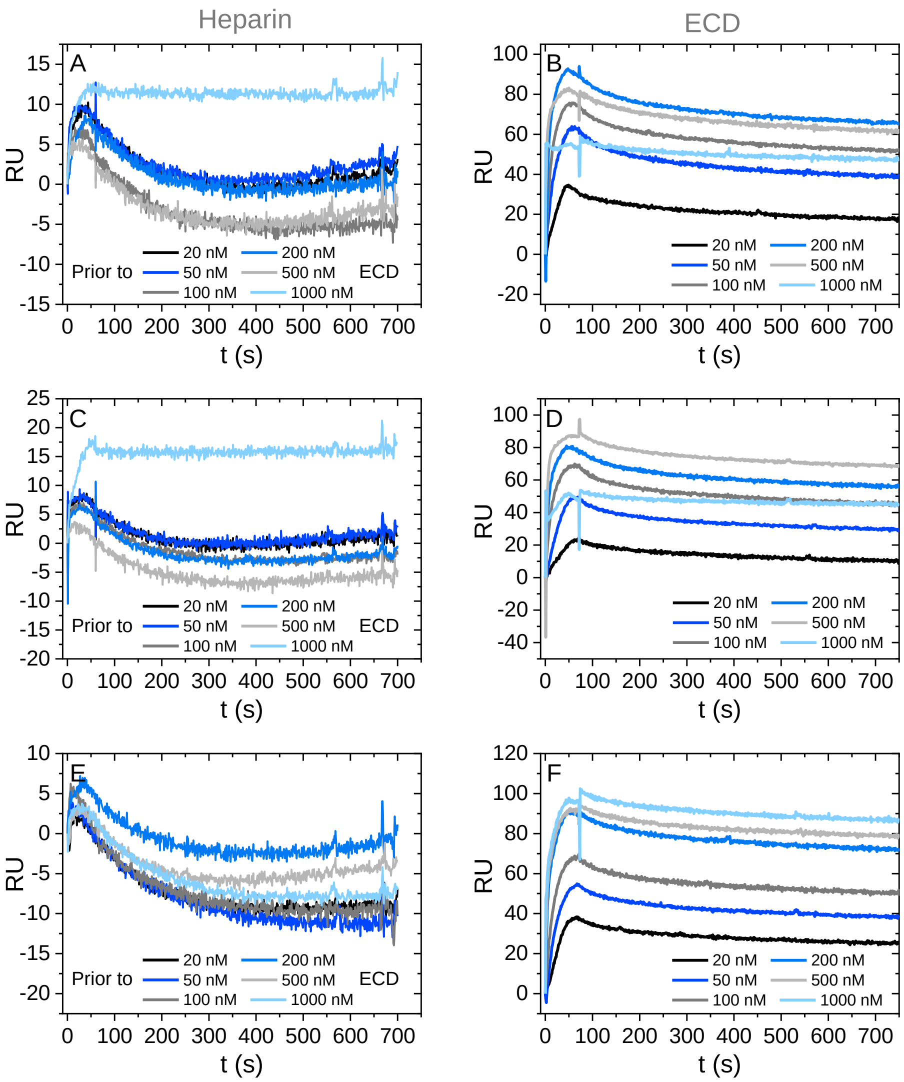

Figure S14. Heparin pre-treatment of immobilized fibronectin increases ECD binding. Heparin (50 µl of 1 µg/ml) was injected at a flow rate of 50 µl/min to a chip with 2 µg/ml immobilized fibronectin (140 ng). Following association, heparin was allowed to dissociate for 10 min, and immediately afterwards, ECD was injected (60 µl at 50 µl/min) at different concentrations: 20 nM, 50 nM, 100 nM, 200 nM, 500 nM, and 1000 nM (the color code is explained in the figure). Following association, ECD was allowed to dissociate for 720 s. The different ECD concentrations were injected following a random order to ensure that the measurements were free of systematic errors, and the progressive increase in ECD binding with increasing concentration reflected the true binding. The surface was regenerated with 2 M NaCl and 0.05 N NaOH after every heparin/ECD binding cycle. The heparin injections are shown in panels (A), (C), and (E), and the corresponding ECD injections in panels (B), (D), and (F). The dose-response experiment was repeated three times using the same chip (run 1: (A), (B); run 2: (C), (D); run3: (E), (F)). Only Fc2-Fc1 traces are shown. Binding of heparin to fibronectin at this concentration did not result in a significant SPR signal (less than 20 RU for all injections). Occasionally, the sensogram line was not stable and drifted to negative values during the long dissociation phase following the heparin treatment. However, there was no correlation between the RU values following heparin treatment and ECD binding. Mean and standard deviation values were calculated for ECD binding from the triplicate experiments. The error associated with each data point was 6-29% (standard deviation normalized against the mean; see Fig. S18).

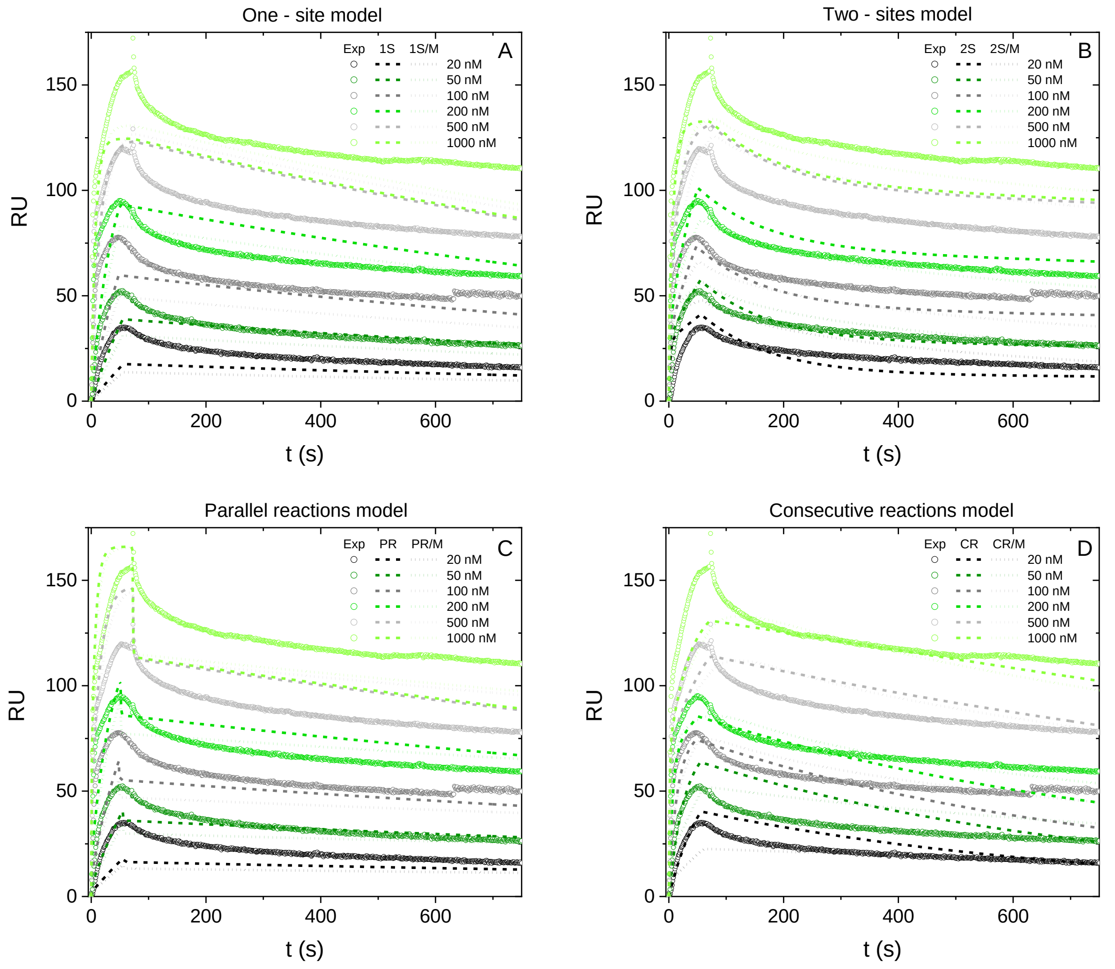

Figure S15. Model fitting of the association and dissociation data for VEGF binding to fibronectin. VEGF was injected (60 µl at 50 µl/min) at different concentrations (20 nM, 50 nM, 100 nM, 200 nM, 500 nM, and 1000 nM; color code explained in the figure) on a chip with 2 µg/ml immobilized fibronectin (140 ng) and pre-treated with heparin (50 µl of 1 µg/ml at 50 µl/min, followed by 10 min dissociation). The data represent mean values of triplicate experiments. The data were fitted with different models describing the association between VEGF and fibronectin (defined in Fig. 6): one-site (1S) (A), two-sites (2S) (B), parallel reactions (PR) (C), and consecutive reactions (CR) (D), considering rapid mixing (dashed lines) or mass transfer (dotted lines; M) phenomena. Model fitting was performed with the beginning of the dissociation time set to the time corresponding to the maximum RU value achieved during the association phase (variable dissociation time). The experimental data are represented by the empty symbols. Best fits were achieved with the two-sites model considering mass transfer. The same dataset and best fit are presented in Fig. 5C.

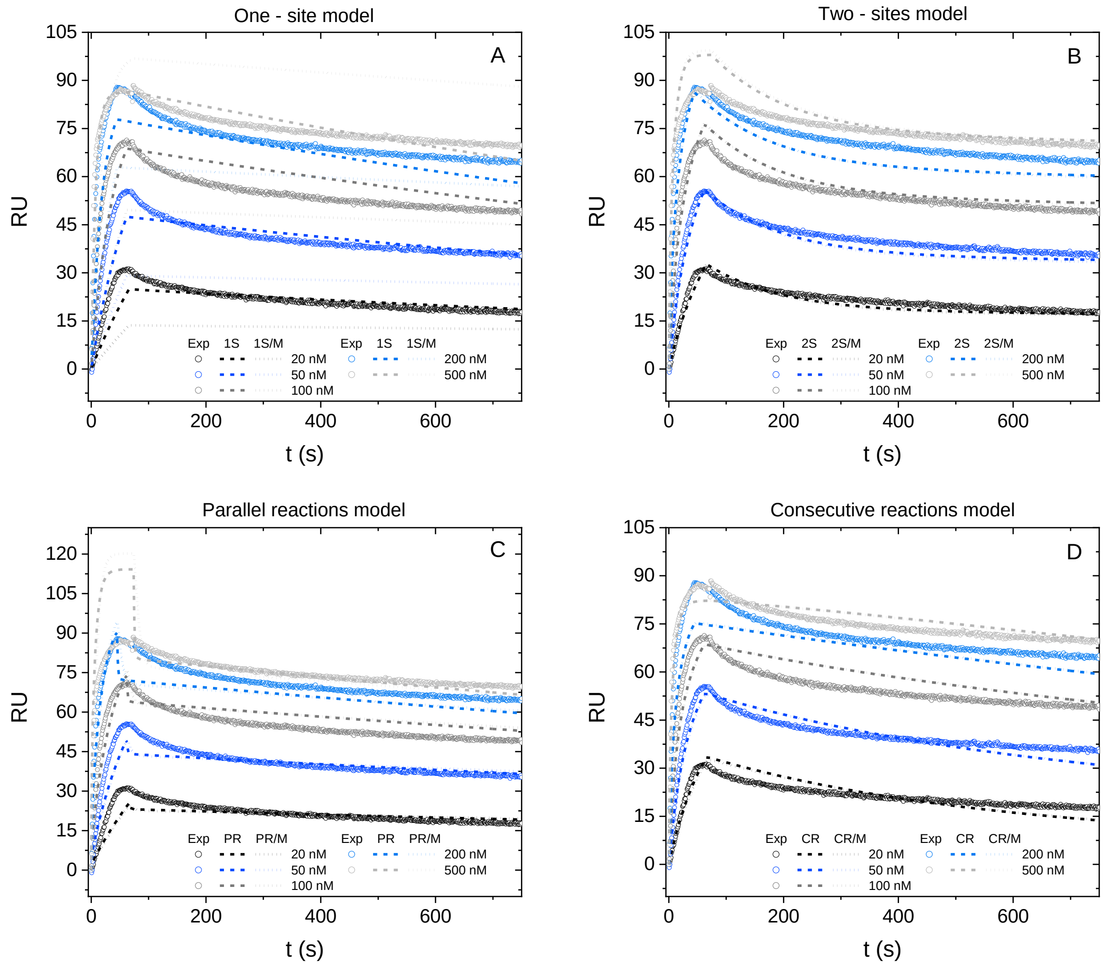

Figure S16. Model fitting of the association and dissociation data for ECD binding to fibronectin. ECD was injected (60 µl at 50 µl/min) at different concentrations (20 nM, 50 nM, 100 nM, 200 nM, and 500 nM; color code explained in the figure) on a chip with 2 µg/ml immobilized fibronectin (140 ng) and pre-treated with heparin (50 µl of 1 µg/ml at 50 µl/min, followed by 10 min dissociation). The data represent mean values of triplicate experiments. The data were fitted with different models describing the association between ECD and fibronectin (defined in Fig. 6): one-site (1S) (A), two-sites (2S) (B), parallel reactions (PR) (C), and consecutive reactions (CR) (D), considering rapid mixing (dashed lines) or mass transfer (dotted lines; M) phenomena. Model fitting was performed with the beginning of the dissociation time set to the time corresponding to the maximum RU value achieved during the association phase (variable dissociation time). The experimental data are represented by the empty symbols. Best fits were achieved with the two-sites model considering rapid mixing. The same dataset and best fit are presented in Fig. 5D.

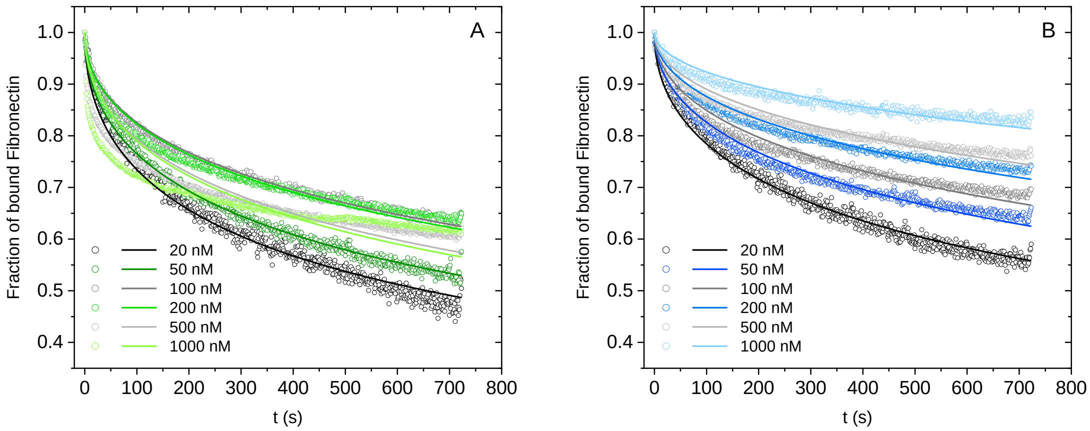

Figure S17. Model fitting of the dissociation data only for VEGF or ECD binding to fibronectin considering rebinding. VEGF (A) or ECD (B) were injected (60 µl at 50 µl/min) at different concentrations (20 nM, 50 nM, 100 nM, 200 nM, 500 nM, and 1000 nM; color code explained in the figure) on a chip with 2 µg/ml immobilized fibronectin (140 ng) and pre-treated with heparin (50 µl of 1 µg/ml at 50 µl/min, followed by 10 min dissociation). Only the dissociation curves were considered. The data represent mean values of triplicate experiments. The RU values were converted to values of the fraction of bound fibronectin (ρ) by normalizing against the initial value at the beginning of dissociation (72 s), which was considered equal to 1. The dissociation data (circles) were fitted to a one-site model taking into account rebinding effects (solid lines), according to the following equation:

$$\rho(t) = \rho(0) * e^{c*t} * erfc(\sqrt{c*t})$$

where:

$$erfc(z) = \frac{2}{\sqrt{\pi}} * \int_{z}^{\infty} e^{-x^2} dx$$

However, the curves showed a clear dependence of $\rho(t)$ on VEGF or ECD concentration. Therefore, the data cannot be fit with a single c parameter. Moreover, several of the individual curves could not be fit at all by this model, especially for high VEGF concentrations. These results further confirm the inability of a one-site model to describe the interactions even if rebinding phenomena are taken into account.

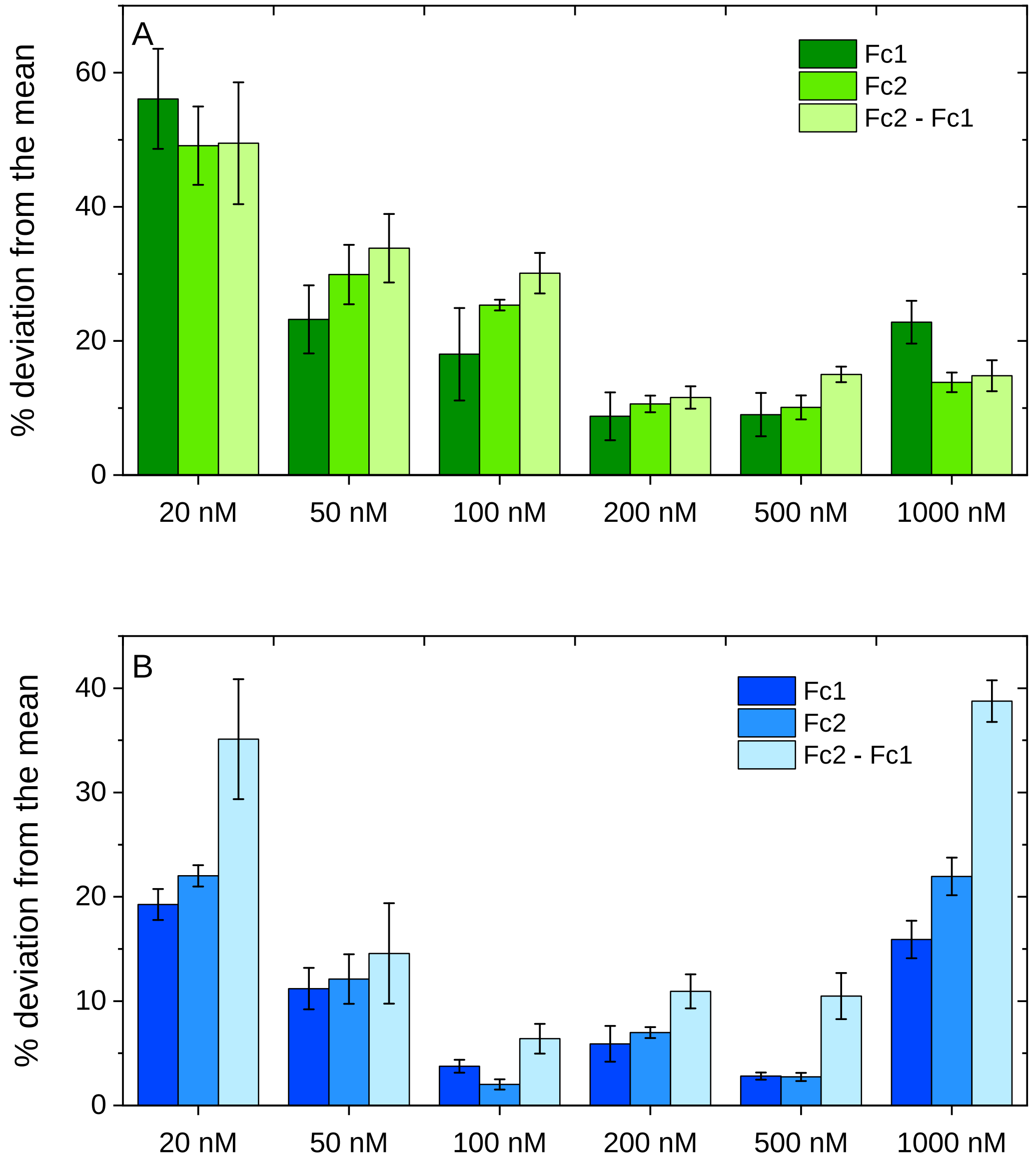

Figure S18. Experimental error associated with SPR dose-response curves. VEGF (A) or ECD (B) were injected (60 µl at 50 µl/min) at different concentrations (20 nM, 50 nM, 100 nM, 200 nM, 500 nM, and 1000 nM) on a chip with 2 µg/ml immobilized fibronectin (140 ng) and pre-treated with heparin (50 µl of 1 µg/ml at 50 µl/min, followed by 10 min dissociation). Each concentration was measured three times on the same chip. Mean and standard deviation values were calculated from the triplicate experiments. For each curve point, the standard deviation was divided by the corresponding mean value to calculate percent errors. A mean percent error value was then calculated from all points of each concentration curve, and are shown for Fc1, Fc2, and Fc2-Fc1.

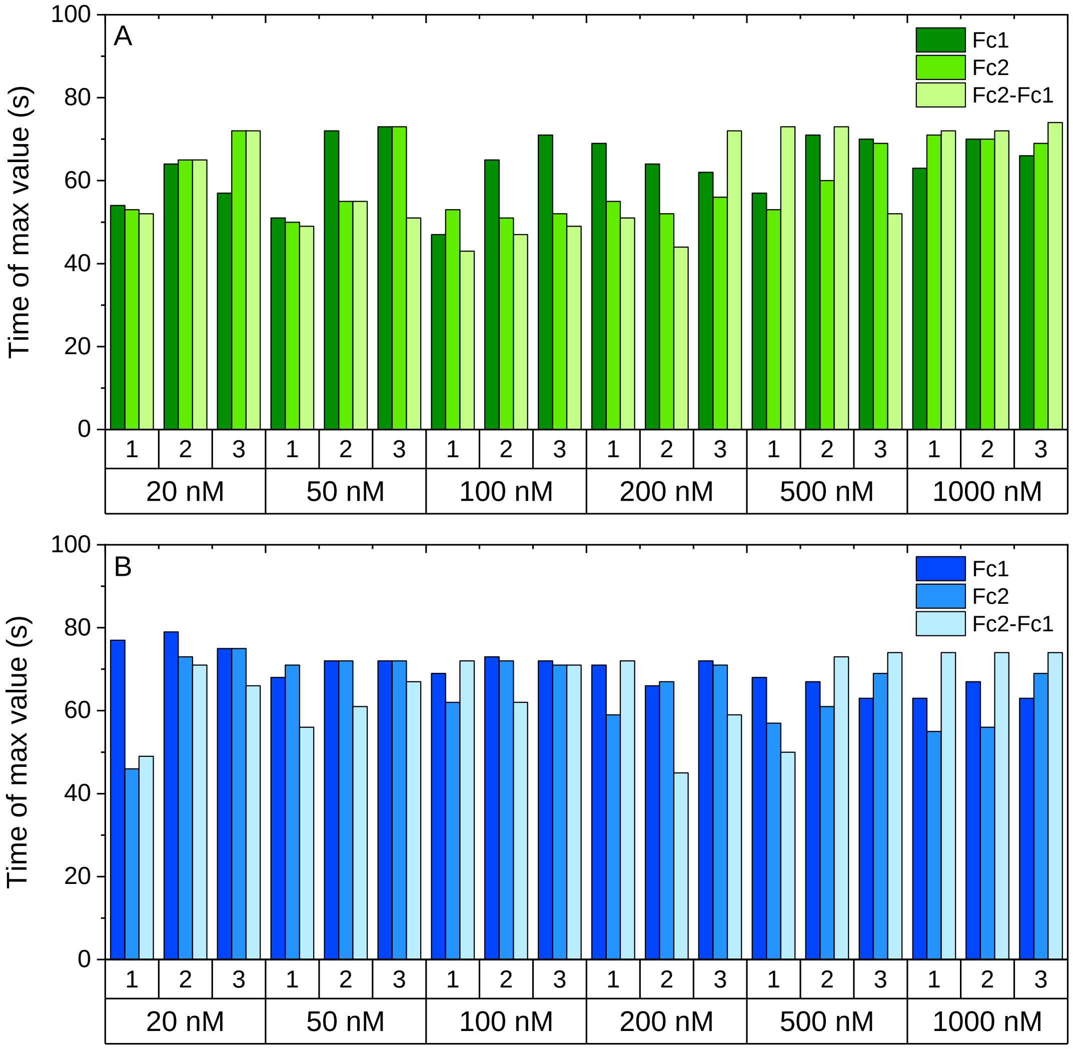

Figure S19. Variation in the time point at which the SPR signal decreases prior to the beginning of the dissociation phase. VEGF (A) or ECD (B) were injected (60 µl at 50 µl/min) at different concentrations (20 nM, 50 nM, 100 nM, 200 nM, 500 nM, and 1000 nM) on a chip with 2 µg/ml immobilized fibronectin (140 ng) and pre-treated with heparin (50 µl of 1 µg/ml at 50 µl/min, followed by 10 min dissociation). Each concentration was measured three times on the same chip. We observed that the RU values started decreasing slightly prior to the nominal beginning of the dissociation phase (72 s). The time point at which RU reached its maximum value during the association phase was calculated and is shown for Fc1, Fc2, and Fc2-Fc1 (color code explained in the figure), for each VEGF and ECD concentration and experimental run (1, 2, 3). The time of maximum RU did not show any obvious correlation with VEGF or ECD concentration, and showed significant variability across replicate experiments, as well as between Fc1 and Fc2 cells. These observations suggest that this variability is likely linked to experimental errors and does not reflect a true aspect of the interaction between fibronectin and VEGF or ECD.

V. Recombinant protein characterization (Figure S20)

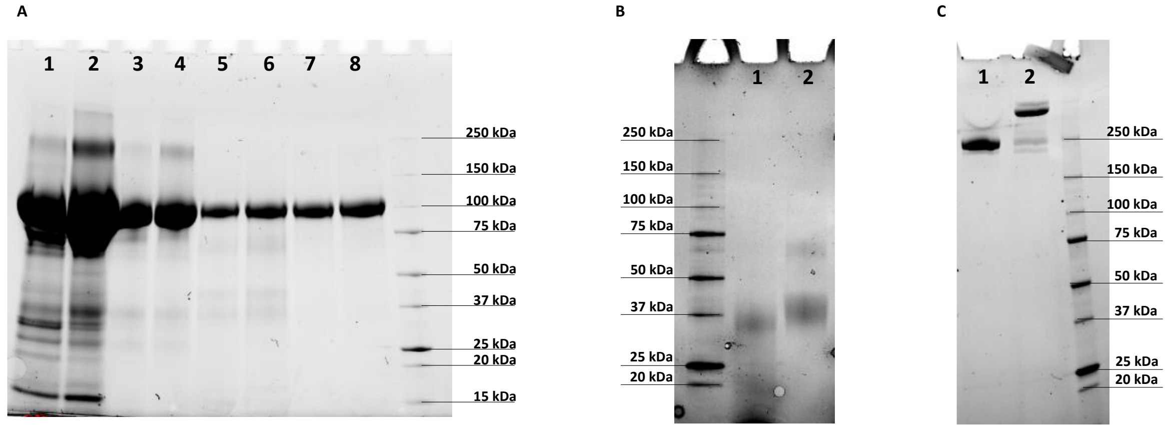

Figure S20. SDS-PAGE analysis of ECD, VEGF, and fibronectin. (A) Recombinant human ECD containing a C-terminal 6x His-tag and expressed in the Sf21 cell line was purified sequentially by immobilized ion affinity chromatography (lanes 1, 2), ion exchange chromatography (lanes 3, 4), and size exclusion chromatography (lanes 5-8). The protein eluted from the final chromatographic step (size exclusion chromatography) was stable after storage at -80 °C (lanes 7, 8), whereas it exhibited slight fragmentation after a 15-day storage period at 4 °C (lanes 5, 6). Two different fractions were collected after immobilized ion affinity chromatography and were processed independently (fraction 1: lanes 1, 3, 5, 7; fraction 2: lanes 2, 4, 6, 8) but resulted in samples indistinguishable by SDS-PAGE. (B) VEGF containing an N-terminal 6x His tag, expressed in *Pichia Pastoris*, and purified by immobilized metal affinity and size exclusion chromatography was analyzed by SDS-PAGE under reducing (lane 1) and non-reducing (lane 2) conditions to ensure sufficient purity and an intact dimeric form. (C). Human plasma fibronectin obtained from Millipore was analyzed by SDS-PAGE under reducing (lane 1) and non-reducing (lane 2) conditions to ensure sufficient purity and an intact dimeric form.

Table S1. Correction factors for adsorption of VEGF and ECD on polystyrene plates from buffers of different ionic strength. Correction factors were calculated from the slopes of the calibration curves shown in Fig. S6 C and D for VEGF and ECD respectively, in order to normalize release data from different extraction buffers to the values corresponding to 150 mM NaCl.

	**VEGF**	**ECD**
**150 mM NaCl**	1.00	1.00
**250 mM NaCl**	0.46	1.03
**500 mM NaCl**	0.44	1.36
**1 M NaCl**	0.18	1.19
**2 M NaCl**	0.19	1.67
**5 M NaCl**	0.86	2.84

Table S2. Free parameter estimation for all models used to fit the association and dissociation data for VEGF and ECD binding to fibronectin. The experimental data (mean values of triplicate measurements) presented on Fig. 5 C, D (as well as Figs. S13-14) were fitted with all possible models shown in Fig. 6, under conditions of rapid mixing or mass transfer, with the beginning of the dissociation phase set to the time corresponding to the maximum RU reached during the association phase (variable t). For each model, the fitting was repeated multiple times with a different set of estimates for the free parameters (different by several orders of magnitude) to reduce as much as possible the danger of the fitting algorithm being trapped in a local minimum. Only solutions where all parameter estimates had a well-defined confidence interval (i.e. confidence intervals that did not contain negative values) were accepted. The solution with the lowest residual sum of squares (RMS) was selected, and the parameter estimates together with their confidence intervals (lower and upper bound) are presented here.

		parameter estimate		lower bound		upper bound	
		rapid mixing	mass transfer	rapid mixing	mass transfer	rapid mixing	mass transfer
VEGF One-site	k_a ($M^{-1}s^{-1}$)	1.38E+05	1.00E+05	1.34E+05	9.63E+04	1.41E+05	1.04E+05
	R_T (mol^1m^{-2})	2.78E-09	2.92E-09	2.75E-09	2.90E-09	2.81E-09	2.94E-09
	k_d (s^{-1})	5.34E-04	5.00E-04	5.10E-04	4.79E-04	5.57E-04	5.20E-04
	k_m (m^1s^{-1})		2.00E-03		1.80E-03		2.19E-03
ECD One-site	k_a ($M^{-1}s^{-1}$)	2.61E+05	1.14E+05	2.57E+05	1.11E+05	2.65E+05	1.17E+05
	R_T (mol^1m^{-2})	1.04E-09	1.19E-09	1.04E-09	1.17E-09	1.05E-09	1.21E-09
	k_d (s^{-1})	4.19E-04	1.40E-04	4.07E-04	1.06E-04	4.31E-04	1.75E-04
	k_m (m^1s^{-1})		2.00E-03		9.37E-04		3.06E-03
VEGF Two-sites	k_{a1} ($M^{-1}s^{-1}$)	2.00E+07	2.00E+07	5.07E+06	9.05E+06	3.49E+07	3.10E+07
	k_{a2} ($M^{-1}s^{-1}$)	1.17E+05	6.50E+04	1.14E+05	6.28E+04	1.20E+05	6.72E+04
	R_T (mol^1m^{-2})	2.95E-09	3.00E-09	2.92E-09	2.97E-09	2.98E-09	3.03E-09
	α	2.19E-01	3.30E-01	2.09E-01	3.18E-01	2.30E-01	3.42E-01
	k_{d1} (s^{-1})	8.29E-03	6.43E-03	7.43E-03	2.87E-03	9.16E-03	9.99E-03
	k_{d2} (s^{-1})	1.23E-04	1.20E-04	8.67E-05	5.28E-05	1.59E-04	1.87E-04
	k_m (m^1s^{-1})		2.95E-06		2.18E-06		3.72E-06
ECD Two-sites	k_{a1} ($M^{-1}s^{-1}$)	1.00E+06	1.00E+06	8.77E+05	8.82E+05	1.12E+06	1.12E+06
	k_{a2} ($M^{-1}s^{-1}$)	2.10E+05	2.04E+05	2.07E+05	2.02E+05	2.13E+05	2.07E+05
	R_T (mol^1m^{-2})	1.19E-09	1.20E-09	1.18E-09	1.20E-09	1.19E-09	1.21E-09
	α	2.48E-01	2.48E-01	2.34E-01	2.34E-01	2.61E-01	2.62E-01
	k_{d1} (s^{-1})	7.60E-03	7.82E-03	7.02E-03	7.19E-03	8.19E-03	8.44E-03

	**k_{d2} (s^{-1})**	5.99E-05	7.82E-05	2.80E-05	4.50E-05	9.18E-05	1.11E-04
	**k_m (m^1s^{-1})**		1.99E-03		6.06E-04		3.38E-03
**VEGF Parallel reactions**	**k_{a1} ($M^{-1}s^{-1}$)**	1.00E+06	1.00E+06	3.09E+05	1.53E+05	1.69E+06	1.85E+06
	**k_{a2} ($M^{-1}s^{-1}$)**	1.40E+05	1.10E+05	1.36E+05	1.07E+05	1.44E+05	1.13E+05
	**R_T (mol^1m^{-2})**	5.07E-09	5.13E-09	5.00E-09	5.10E-09	5.13E-09	5.15E-09
	**k_{d1} (s^{-1})**	1.18E+00	1.29E+00	3.58E-01	1.89E-01	2.00E+00	2.39E+00
	**k_{d2} (s^{-1})**	3.59E-04	2.46E-04	3.35E-04	2.26E-04	3.84E-04	2.66E-04
	**k_m (m^1s^{-1})**		1.99E-03		1.83E-03		2.15E-03
**ECD Parallel reactions**	**k_{a1} ($M^{-1}s^{-1}$)**	1.00E+06	1.00E+06	6.56E+05	2.76E+05	1.34E+06	1.72E+06
	**k_{a2} ($M^{-1}s^{-1}$)**	2.60E+05	2.90E+05	2.55E+05	2.83E+05	2.65E+05	2.97E+05
	**R_T (mol^1m^{-2})**	1.94E-09	1.86E-09	1.92E-09	1.85E-09	1.96E-09	1.88E-09
	**k_{d1} (s^{-1})**	6.82E-01	3.97E-01	4.44E-01	1.29E-01	9.19E-01	6.65E-01
	**k_{d2} (s^{-1})**	2.75E-04	2.30E-04	2.60E-04	2.11E-04	2.91E-04	2.48E-04
	**k_m (m^1s^{-1})**		2.35E-06		1.89E-06		2.82E-06
**VEGF Consecutive reactions**	**k_{aR} ($M^{-1}s^{-1}$)**	7.73E+05	1.50E+06	7.10E+05	1.30E+06	8.37E+05	1.70E+06
	**k_{aRA} ($M^{-1}s^{-1}$)**	2.78E+04	1.50E+04	2.60E+04	1.32E+04	2.96E+04	1.68E+04
	**R_T (mol^1m^{-2})**	1.57E-09	1.74E-09	1.54E-09	1.68E-09	1.61E-09	1.80E-09
	**k_{dRA} (s^{-1})**	1.46E-03	2.83E-03	1.36E-03	2.43E-03	1.55E-03	3.23E-03
	**k_{dR2A} (s^{-1})**	4.53E-04	8.12E-04	4.18E-04	7.47E-04	4.88E-04	8.77E-04
	**k_m (m^1s^{-1})**		3.57E-07		3.34E-07		3.80E-07
**ECD Consecutive reactions**	**k_{aR} ($M^{-1}s^{-1}$)**	1.00E+06	1.00E+06	9.49E+05	9.38E+05	1.05E+06	1.07E+06
	**k_{aRA} ($M^{-1}s^{-1}$)**	2.30E+05	2.30E+05	2.25E+05	2.24E+05	2.35E+05	2.36E+05
	**R_T (mol^1m^{-2})**	4.96E-10	4.96E-10	4.89E-10	4.94E-10	5.03E-10	4.98E-10
	**k_{dRA} (s^{-1})**	2.13E-03	2.15E-03	1.99E-03	1.98E-03	2.28E-03	2.32E-03
	**k_{dR2A} (s^{-1})**	3.24E-04	3.41E-04	3.08E-04	3.24E-04	3.41E-04	3.58E-04
	**k_m (m^1s^{-1})**		4.62E-06		1.01E-06		8.23E-06
